# Checklist of Korean Mymaridae (Hymenoptera, Chalcidoidea) with new records and descriptions of two new species of *Zeyanus* from South Korea and Japan

**DOI:** 10.3897/zookeys.1295.200780

**Published:** 2026-10-21

**Authors:** Serguei V. Triapitsyn, Soohyun Kim, Ilgoo Kang

**Affiliations:** 1 Entomology Research Museum, Department of Entomology, University of California, Riverside, California 92521, USA Entomology Research Museum, Department of Entomology, University of California Riverside United States of America; 2 Department of Ecological Science, Kyungpook National University, 2559 Gyeongsang-daero, Sangju, Gyeongsangbuk-do, 37224, Republic of Korea Department of Ecological Science, Kyungpook National University Sangju Republic of Korea; 3 Department of Entomology, Kyungpook National University, 2559 Gyeongsang-daero, Sangju, Gyeongsangbuk-do, 37224, Republic of Korea Department of Entomology, Kyungpook National University Sangju Republic of Korea; 4 Research Institute of Invertebrate Vector, Kyungpook National University, 2559 Gyeongsang-daero, Sangju, Gyeongsangbuk-do, 37224, Republic of Korea Research Institute of Invertebrate Vector, Kyungpook National University Sangju Republic of Korea

**Keywords:** Biodiversity, *
Dicopomorpha
*, fairyfly, Korean Peninsula, *
Mymar
*

## Abstract

A checklist of Mymaridae (Hymenoptera, Chalcidoidea) in South Korea is presented, comprising the previously recorded 15 genera and 31 species and also the five newly recorded taxa, totaling 36 species. Two new species of *Zeyanus* Huber, *Z. knuz* Triapitsyn & Kim, **sp. nov**. and *Z. sunayez* Triapitsyn & Kim, **sp. nov**., are described and illustrated from South Korea and Japan. An updated key to females of *Zeyanus* species is provided. Newly recorded from South Korea are *Mymar ermak* Triapitsyn & Berezovskiy, *M. maritimum* Triapitsyn & Berezovskiy, and *M. pulchellum* Curtis. *Dicopomorpha* Ogloblin and *D. koreana* Triapitsyn & Berezovskiy are reported for the first time from Taiwan (China). COI sequences are obtained for three species of *Mymar* Curtis (*M. ermak*, *M. maritimum*, *M. pulchellum*) as well as for *Zeyanus sunayez* as a contribution to building a genetic library of fairyflies.

## Introduction

Fairyflies (Hymenoptera, Mymaridae) have a short history of study in the Korean Peninsula, with 15 genera and 31 species known so far from South Korea only. Most of these were included in regional revisions of certain genera in the Palearctic region (Triapitsyn 2003, 2010, 2013, 2021, 2023; Triapitsyn and Berezovskiy 2003, 2004a, 2004b). These scattered records are summarized in the checklist below, the first such account for the country. It serves as a starting point of an upcoming comprehensive study on biodiversity of Mymaridae in South Korea, which begins with this assessment of previously available records and an inventory of specimens in the insect collections within and outside of the country.

Unfortunately, only a few specimens of Korean Mymaridae are available for study besides those in the largest insect collections: the Korea National Arboretum (Pocheon-si, South Korea) and the University of California at Davis and Riverside (USA). Many voucher specimens of the previously recorded mymarid genera and species have been lost, including egg parasitoids associated with rice-feeding leafhoppers and planthoppers (Hemiptera, Cicadellidae and Delphacidae, respectively), some of which were also misidentified.

Paik (1974a) published the first records of Korean Mymaridae as a short list of five genera: *Anagrus* Haliday (two unidentified species), *Gonatocerus* Nees ab Esenbeck (one unidentified species), *Ooctonus* Haliday, *Mymar* Curtis (one unidentified species), and *Polynema* Haliday (one unidentified species). However, no voucher specimens of that review article could be located; all of these were assumed to be natural enemies of cicadellids in rice fields. Paik (1978) listed three identified and three unidentified species of Korean Mymaridae in five genera. Sahad and Hirashima (1984) studied specimens of *Anagrus* and *Gonatocerus* collected in South Korea in the 1970s and the early 1980s (mainly from Suwon-si, Gyeonggi-do and Miryang-si, Gyeongsangnam-do), without any host information being provided; these were captured by J.C. [Jong Cheol] Paik. Lamentably, those identified voucher specimens are also lost (the first author of this communication could not find them among the K.A. Sahad specimens during several visits to the insect collection of Entomological Laboratory, Faculty of Agriculture, Kyushu University, Fukuoka, Japan [ELKU]). Most of the previous and subsequent identifications and records of the Korean Mymaridae in the 1980s and 1990s were also of the egg parasitoids of rice cicadellids and delphacids (Chang 1980; Kim et al. 1982; Yeo et al. 1990; Chang et al. 1991). However, reliable host records are known only for a few positively identified species of Mymaridae from South Korea (e.g., Triapitsyn et al. 2026), as indicated in the checklist below. The list now includes 15 genera and 36 species, and undoubtedly it will be further expanded in the future.

Here we also describe two distinctive new species of *Zeyanus* Huber from South Korea and Japan and provide new records of the three previously described species of *Mymar*, along with their images and molecular data.

## Material and methods

All specimens examined were identified to genus and species by the first author among the South Korean Mymaridae deposited in the Korea National Insect Collection, Korea National Arboretum, Pocheon-si, Republic of Korea **(KNA)**, Department of Entomology, Kyungpook National University, Sangju-si, Republic of Korea **(KNUS)**, R.M. Bohart Museum of Entomology, University of California, Davis, California, USA **(UCDC)**, and Entomology Research Museum, University of California, Riverside, California, USA **(UCRC)**. Additional specimens of *Zeyanus* from Japan were borrowed from the Laboratory of Entomology, Faculty of Agriculture, Meijo University, Tempaku, Nagoya, Japan (**ZLMU**). Specimens are mostly mounted dry on points, but some in KNA and KNUS are stored in ethanol. The holotypes of both new species of *Zeyanus* were slide-mounted using the technique described in Huber (2015) and one of them, originally from the UCDC material, is deposited in the collection of the National Institute of Biological Resources, Incheon, Republic of Korea (**NIBR**). An extralimital specimen of *Dicopomorpha koreana* Triapitsyn & Berezovskiy is deposited in the National Museum of Natural Science, Taichung, Taiwan (China) (**NMNS**). Specimens deposited in KNA and KNUS were collected by Malaise traps, except for *Anagrus atomus* (L.), which was collected by sweeping.

Terms for morphological features mainly follow Gibson (1997), with some modifications used in Triapitsyn et al. (2017), like the incorrect use of “marginal vein” for parastigma, which is reluctantly retained here for consistency and to avoid confusion. All measurements are given in micrometers (µm), either as length or, for the wings, as length: width. Abbreviations used are **F** = funicular segment of the female antenna or flagellar segment of the male antenna and **mps** = multiporous plate sensillum or sensilla on the flagellar segments (= longitudinal sensillum or sensilla or sensory ridge(s) of some authors). Images of dried or ethanol-preserved specimens were taken using a Leica Z16 microscope (Leica, Switzerland) with a Leica DFC450 camera (Leica, Switzerland), or a Sony A7R4 camera (Sony, Japan), equipped with either a Canon Macro Photo Lens MP-E 65 mm f/2.8 1–5 × (Canon, Japan) or Mitutoyo M Plan APO infinity-corrected microscopic objective lenses (10 ×) (Mitutoyo, Japan). Images of slide mounted specimens were taken using ZEISS Axioskop 2 plus compound microscope. LAS X software (Leica, Switzerland) or Zerene Stacker (Zerene System, USA) were used for image stacking. Adobe Photoshop CS6 (Adobe, USA) was used to edit images and create plates.

A key to the females of all known species of *Zeyanus* was updated from that in Triapitsyn et al. (2017).

To contribute to a genetic library, DNA was extracted non-destructively from individual females of the three *Mymar* species as well as from a female paratype of *Zeyanus sunayez* sp. nov. using the DNeasy Blood and Tissue Kit (Qiagen, Germany) following the manufacturer’s instructions. The extracted specimens were subsequently slide-mounted. All DNA samples were stored at -22 °C until further analysis. Mitochondrial cytochrome *c* oxidase subunit I (COI) gene was targeted for polymerase chain reaction (PCR). The COI gene was amplified using the following primer sets: LepF1 (5'-ATTCAACCAATCATAAAGATATTGG-3') and LepR1 (5'-TAAACTTCTGGATGTCCAAAAAATC A-3') (Hebert et al. 2004) or BeeCox1F1 (TAGTCAACAAATCATAAAGATATTGG) and BeeCox1R2 (CCAAATCCTGGTAGAATTAAAATATA) (Bleidorn and Henze 2021). PCR conditions consisted of an initial denaturation at 94 °C for 3 min, followed by 5 cycles of 94 °C for 30 s, 45 °C for 25 s, and 68 ° C for 1 min; 35 additional cycles of 94 °C for 30 s, 50 °C for 25 s, and 68 °C for 1 min; and a final extension at 68 °C for 7 min. Amplified PCR products were cleaned up and sequenced at the Macrogen Inc. (Sejong, South Korea) using the 3730xl DNA Analyzer (Applied Biosystems, Foster City, CA, USA). The obtained DNA sequences were edited and assembled using Geneious Prime 2025.2 (https://www.geneious.com) or Bioedit (Hall 1999).

## Checklist of genera and species of Mymaridae previously recorded from South Korea (in alphabetical order)

### 1. Anagroidea marina

Taxon classificationAnimaliaHymenopteraMymaridae

Triapitsyn & Berezovskiy, 2002

4E4F855D-10E2-5CA2-B809-2F22D817112C

Anagroidea marina Triapitsyn & Berezovskiy, 2002: 8 (original description).

#### Distribution.

Oriental region: China: Yunnan (Jin and Li 2016) and Taiwan (Triapitsyn 2018); Palearctic region: China (Sichuan), Japan, Russia (Far East), South Korea (Triapitsyn and Berezovskiy 2002; Triapitsyn 2018).

#### Korean records.

*Anagroidea marina* Triapitsyn & Berezovskiy: Triapitsyn 2018: 118 (Gangwon-do).

### 2. Anagrus (Anagrus) arboridiae

Taxon classificationAnimaliaHymenopteraMymaridae

Triapitsyn & Adachi-Hagimori, 2020

C9E7C95D-F9C9-5612-AB0A-CB675A4A8B2A

Anagrus (Anagrus) arboridiae Triapitsyn & Adachi-Hagimori in Triapitsyn et al. 2020: 135 (original description).

#### Distribution.

Palearctic region: Japan (Triapitsyn et al. 2020), South Korea (Triapitsyn et al. 2026).

#### Korean records.

Anagrus (Anagrus) arboridiae Triapitsyn & Adachi-Hagimori: Triapitsyn et al. 2026: 170–173 (Chungcheongbuk-do: Okcheon-gun, Samcheong-ri; hosts: *Arboridia maculifrons* (Vilbaste) and *Arboridia* sp. [Cicadellidae]).

### 3. Anagrus (Anagrus) atomus

Taxon classificationAnimaliaHymenopteraMymaridae

(Linnaeus, 1767)

17225F8B-EC1E-53F4-9CA6-80BE9413AE8F

Ichneumon atomus Linnaeus, 1767: 941 (original description).
Anagrus (Anagrus) atomus (L.): Triapitsyn 2015a: 19–20, 41 (coded redescription, distribution, hosts, list of synonyms); Triapitsyn et al. 2020: 1743–1752 (taxonomic history, synonyms, diagnosis, distribution, hosts, molecular identification).

#### Distribution.

Afrotropical region: Cape Verde, Yemen; Australasia: New Zealand; Nearctic region: Canada, Mexico, USA; Neotropical region: Argentina, Chile; Oriental region: China, Pakistan; Palearctic region: throughout (Triapitsyn 2015a). Probably non-native (inadvertently introduced) in Australasia and the Neotropical and Nearctic regions (Triapitsyn et al. 2020).

#### Korean records.

Anagrus (Anagrus) atomus (Linnaeus): Triapitsyn and Berezovskiy 2004a: 13–14 (Jeju-do); Triapitsyn 2015a: 20.

#### Material examined.

South Korea • 1 ♀; Chungcheongnam-do, Dangjin-si, Myeoncheon-myeon, 36°49.15'N, 126°39.95'E, alt. 62 m, 14.vii.2025, S.H. Kim, J.H. Kim; KNUS.

### 4. Anagrus (Anagrus) flaviapex

Taxon classificationAnimaliaHymenopteraMymaridae

Chiappini & Lin, 1998

9FF1E99B-DF32-5FAA-A2B9-785C8BBE4F1C

Anagrus (Anagrus) flaviapex Chiappini & Lin, 1998: 562 (original description).
Anagrus (Anagrus) flaviapex Chiappini & Lin: Triapitsyn 2015a: 22 (coded redescription, distribution, hosts).

#### Distribution.

Oriental region: Australia (Christmas Island), Cambodia, China, India, Japan (Okinawa Island), Thailand; Palearctic region: South Korea (Triapitsyn 2015a; Triapitsyn et al. 2020).

#### Korean records.

Anagrus (Anagrus) flaviapex Chiappini & Lin: Triapitsyn 2015a: 22 (Gyeonggi-do).

### 5. Anagrus (Anagrus) hirashimai

Taxon classificationAnimaliaHymenopteraMymaridae

Sahad, 1982

B251CDE6-FA71-5651-9591-6325044C4C63

Anagrus hirashimai Sahad, 1982: 198 (original description).
Anagrus (Anagrella) hirashimai Sahad: Triapitsyn 2015a: 16 (coded redescription, distribution).

#### Distribution.

Oriental region: China (Chiappini and Lin 1998); Palearctic region: Japan (Sahad 1982), Russia (Far East), South Korea (Triapitsyn 2015a).

#### Korean records.

Anagrus (Anagrella) hirashimai Sahad: Triapitsyn and Berezovskiy 2004a: 5 (Gyeonggi-do); Triapitsyn 2015a: 16.

### 6. Anagrus (Anagrus) incarnatus

Taxon classificationAnimaliaHymenopteraMymaridae

Haliday, 1833

BF23F2FB-6B6A-505C-AB1D-3B5B4179593C

Anagrus incarnatus Haliday, 1833: 347 (original description).
Anagrus (Anagrus) incarnatus Haliday: Triapitsyn et al. 2018: 2802–2814 (taxonomic history, synonyms, redescription, distribution, hosts, molecular identification).

#### Distribution.

Afrotropical region: Mauritius; Australasia: New Zealand; Nearctic region: Canada, Mexico, USA; Neotropical Region: Argentina; Oceania: Hawaiian Islands, Northern Mariana Islands (Guam); Oriental region: almost throughout; Palearctic region: throughout (Triapitsyn et al. 2018).

#### Korean records.

*Anagrus incarnatus* Haliday: Sahad and Hirashima 1984: 3, 46–50 (Gyeongsangnam-do); Yeo et al. 1990: 1–5 (laboratory reared, either in Daejeon or Gyeonggi-do; host: *Nilaparvata lugens* (Stål) [Delphacidae] on rice plants); Chang et al. 1991: 102–104 (laboratory reared, either in Daejeon or Gyeonggi-do; host: *Nilaparvata lugens* on rice plants); Lee et al. 2021: 699 (list).

*Anagrus nigriventris* Girault: Sahad and Hirashima 1984: 3, 52–55 (Gyeonggi-do, as *A.* ?nigriventris); Lee et al. 2021: 699 (list). Misidentified, because *A. nigriventris* is a strictly New World species (Triapitsyn 2015a); most likely (but not certainly) it was *A. incarnatus*.

*Anagrus flaveolus* Waterhouse: Sahad and Hirashima 1984: 3, 55–58 (Gyeonggi-do); Lee et al. 2021: 699 (list). Misidentified, because *A. flaveolus* is strictly a New World species (Triapitsyn 2015a).

*Anagrus* [sp.] nr flaveolus [Waterhouse]: Kim et al. 1979: 570–579 (Gyeongsangnam-do: Jinju-si; host: *Laodelphax striatellus* (Fallén) [Delphacidae], *Nephotettix cincticeps* Uhler [Cicadellidae]); Kim et al. 1982: 88–93 (Gyeongsangnam-do; hosts: *Laodelphax striatellus*, *Nilaparvata lugens*, and *Sogatella furcifera* (Horváth) [Delphacidae] on rice plants).

Anagrus (Anagrus) nilaparvatae Pang & Wang: Triapitsyn and Berezovskiy 2004a: 21–22 (Gangwon-do, Gyeonggi-do); Triapitsyn 2015a: 36.

Anagrus (Anagrus) incarnatus Haliday: Triapitsyn et al. 2018: 2813.

#### Material examined.

South Korea • 1 ♀; Gyeongsangbuk-do, Chilgok-gun, Gisan-myeon, 35°59.07'N, 128°21.20'E, alt. 78 m, 12.x.–9.xii.2024, S.H. Kim; KNUS.

### 7. Anagrus (Anagrus) obscurus

Taxon classificationAnimaliaHymenopteraMymaridae

Förster, 1861

62AB6571-1AE1-558A-B9D9-2A4D4911FDA5

Anagrus obscurus Förster, 1861: 43 (original description).
Anagrus (Anagrus) obscurus Förster: Triapitsyn 2015a: 36 (coded redescription, distribution, hosts).

#### Distribution.

Palearctic region: Austria, Bulgaria, Finland, Greece, Italy, Kyrgyzstan, Northern Macedonia, Russia, South Korea, Spain, Switzerland, UK (England) (Triapitsyn 2015a; Triapitsyn et al. 2020).

#### Korean records.

Anagrus (Anagrus) obscurus Förster: Triapitsyn and Berezovskiy 2004a: 27 (Jeju-do, Gyeonggi-do); Triapitsyn 2015a: 36.

### 8. Anagrus (Paranagrus) optabilis

Taxon classificationAnimaliaHymenopteraMymaridae

(Perkins, 1905)

9A142E0E-EC24-55C4-A93C-0C6A9A440290

Paranagrus optabilis Perkins, 1905: 199 (original description).
Anagrus (Paranagrus) optabilis (Perkins): Triapitsyn 2015a: 17 (coded redescription, distribution, hosts).

#### Distribution.

Afrotropical region: Cape Verde, Madagascar, Mauritius, Réunion, South Africa, Yemen; Australasia: Australia, New Zealand, Papua New Guinea; Nearctic region: USA; Oceania: Fiji, Hawaiian Islands, Northern Mariana Islands (Guam), Samoa; Oriental region: Bangladesh, China (including Taiwan), India, Indonesia, Malaysia, Philippines, Sri Lanka, Thailand; Palearctic region: Canary Islands, China, Finland, Japan, Madeira, Russia (Far East), South Korea, Spain (Triapitsyn 2015a; Triapitsyn et al. 2020).

#### Korean records.

*Anagrus optabilis* (Perkins): Sahad and Hirashima 1984: 3, 65–68 (Gyeonggi-do, Gyeongsangnam-do); Lee et al. 2021: 699 (list).

Anagrus (Paranagrus) optabilis (Perkins): Triapitsyn 2015a: 17.

### 9. Anagrus (Anagrus) striatus

Taxon classificationAnimaliaHymenopteraMymaridae

Chiappini & Lin, 1998

2F15F4B9-1D73-5A3A-99F5-E22DEE2F78DD

Anagrus (Anagrus) striatus Chiappini & Lin, 1998: 566 (original description).
Anagrus (Anagrus) striatus Chiappini & Lin: Triapitsyn 2015a: 37 (coded redescription, distribution).

#### Distribution.

Oriental Region: China (Chiappini and Lin 1998); Palearctic region: China (Chiappini and Lin 1998), South Korea (Triapitsyn and Berezovskiy 2004a).

#### Korean records.

Anagrus (Anagrus) striatus Chiappini & Lin: Triapitsyn and Berezovskiy 2004a: 31 (Gyeonggi-do); Triapitsyn 2015a: 37.

### 10. Anaphes (Patasson) collinus

Taxon classificationAnimaliaHymenopteraMymaridae

Walker, 1846

C902E606-A8A3-5F6E-B0F9-BB67EB0FBD28

Anaphes collinus Walker, 1846: 52 (original description).
Anaphes (Patasson) collinus Walker: Triapitsyn 2023: 158–160 (taxonomic history, diagnosis, illustrations, distribution, hosts); Thuróczy 2025: 96–107 (taxonomic history, synonyms, diagnosis, illustrations, distribution, hosts).

#### Distribution.

Nearctic region: Canada, USA; Palearctic region: Austria, Belgium, China, Czechia, Denmark, Finland, France, Germany, Hungary, Ireland, Italy, Kyrgyzstan, Netherlands, Norway, Poland, Romania, Russia, South Korea, Turkmenistan, Ukraine, UK (Triapitsyn 2023; Thuróczy 2025).

#### Korean records.

Anaphes (Patasson) collinus Walker: Triapitsyn 2023: 158 (Gyeonggi-do).

### 11. Anaphes (Anaphes) flavipes

Taxon classificationAnimaliaHymenopteraMymaridae

(Foerster, 1841)

6D4D85AE-333F-53C6-8C7E-CC4153C30B69

Gonatocerus flavipes Foerster, 1841: 45 (original description).
Anaphes (Anaphes) flavipes Foerster: Triapitsyn 2023: 135–137 (taxonomic history, diagnosis, illustrations, distribution, hosts); Thuróczy 2025: 219–236 (taxonomic history, synonyms, diagnosis, illustrations, distribution, hosts).

#### Distribution.

Nearctic region (introduced): Canada, USA; Oriental region: China; Palearctic region: almost throughout (Triapitsyn 2023; Thuróczy 2025).

#### Korean records.

*Anaphes nipponicus* Kuwayama: Paik 1978: 180 (host: *Oulema oryzae* (Kuwayama) [Chrysomelidae]); Lee et al. 2021: 699 (list).

Anaphes (Anaphes) nipponicus Kuwayama: Triapitsyn 2021: 35–36 (Gyeonggi-do).

Anaphes (Anaphes) flavipes (Foerster): Triapitsyn et al. 2023: 10; Triapitsyn 2023: 136.

### 12. Anaphes (Anaphes) gauthieri

Taxon classificationAnimaliaHymenopteraMymaridae

Debauche, 1948

A5B34FBC-C19D-52B8-B0DF-07871955DBB3

Anaphes (Anaphes) gauthieri Debauche, 1948: 171 (original description).
Anaphes (Anaphes) gauthieri Debauche: Triapitsyn 2023: 138–140 (taxonomic history, diagnosis, illustrations, distribution, host); Thuróczy 2025: 251–267 (taxonomic history, synonyms, diagnosis, illustrations, distribution, hosts).

#### Distribution.

Palearctic region: Austria, Belgium, Czechia, Finland, Germany, Italy, Kyrgyzstan, Netherlands, Poland, Romania, Russia, Serbia, South Korea, Sweden, Switzerland, UK (Triapitsyn 2023; Thuróczy 2025). Some of these may need confirmation (Thuróczy 2025).

#### Korean records.

Anaphes (Anaphes) gauthieri Debauche: Triapitsyn 2021: 24 (Gyeonggi-do); Triapitsyn 2023: 139.

#### Remarks.

Thuróczy (2025: 550) commented that Triapitsyn’s (2021) identification of this species from South Korea requires confirmation.

### 13. Anaphes (Anaphes) regulus

Taxon classificationAnimaliaHymenopteraMymaridae

Walker, 1846

CB636DAE-E8B4-556F-A5F5-FDA747D91171

Anaphes regulus Walker, 1846: 52 (original description).
Anaphes (Anaphes) regulus Walker: Triapitsyn 2023: 147–148 (taxonomic history, diagnosis, illustrations, distribution, host); Thuróczy 2025: 534–546 (taxonomic history, synonyms, diagnosis, illustrations, distribution, hosts).

#### Distribution.

Palearctic region: Belgium, Czechia, Denmark, Finland, Germany, Iceland, Ireland, Netherlands, Norway, Poland, Russia, South Korea, UK (Triapitsyn 2023; Thuróczy 2025). Some of these may need confirmation, or were likely misidentified (Thuróczy 2025), although the true identity of this nominal species would inevitably remain in question until DNA is successfully extracted from the dry-mounted lectotype (if that is at all possible) and the resulting sequences are analyzed.

#### Korean records.

Anaphes (Anaphes) regulus Walker: Triapitsyn 2021: 38–39 (Gyeonggi-do); Triapitsyn 2023: 148.

#### Remarks.

Thuróczy (2025: 219) commented that Triapitsyn (2021, 2023) misidentified Anaphes (Anaphes) flavipes as Anaphes (Anaphes) regulus, but that would require further investigation, preferably by a molecular study.

### 14. Dicopomorpha koreana

Taxon classificationAnimaliaHymenopteraMymaridae

Triapitsyn & Berezovskiy, 2003

B680A5AD-7F9A-5CD9-BC2D-88D77F001C31

Dicopomorpha koreana Triapitsyn & Berezovskiy, 2003: 11 (original description).
Dicopomorpha koreana Triapitsyn & Berezovskiy: Pawluk and Painter 2026: 555–557 (taxonomic history, diagnosis, illustrations, distribution).

#### Distribution.

Oriental region: India, Taiwan (China) (new record); Palearctic region: South Korea (Triapitsyn and Berezovskiy 2003; Pawluk and Painter 2026).

#### Korean records.

*Dicopomorpha koreana* Triapitsyn & Berezovskiy: Triapitsyn and Berezovskiy 2003: 11–13 (Gangwon-do); Lee et al. 2021: 699 (list); Pawluk and Painter 2026: 554 (list), 3–4 (key, taxonomic history, diagnosis, distribution, illustrations of the holotype).

#### Material examined.

South Korea • 1 ♀; Gyeongsangnam-do, Changwon-si, Masanhappo-gu, Jinjeon-myeon, 35°06.97'N, 128°24.73'E, alt. 44 m, 30.viii.–24.xi.2024, J.H. Kim; KNUS.

#### Extralimital material examined.

Taiwan (China) • 1 ♀; Nantou County, Xitou Forest, Liolongtou, 23°39'19.2"N, 120°47'14.8"E, alt. 1750 m, 19.vii.–21.viii.2017, H.-T. Yeh; NMNS.

#### Remarks.

This is a new record of the genus *Dicopomorpha* Ogloblin and of *D. koreana* from Taiwan (China).

### 15. Erythmelus (Erythmelus) nuinu

Taxon classificationAnimaliaHymenopteraMymaridae

Triapitsyn, 2003

9E6B02AA-2070-559F-B26D-E6BED7BA2B05

Erythmelus (Erythmelus) nuinu Triapitsyn, 2003: 30 (original description).

#### Distribution.

Oriental region: India (Manickavasagam et al. 2011); Palearctic region: China, Russia (Far East), South Korea (Triapitsyn 2003).

#### Korean records.

Erythmelus (Erythmelus) nuinu Triapitsyn: Triapitsyn 2003: 30–33 (Gangwon-do).

### 16. Eubroncus prodigiosus

Taxon classificationAnimaliaHymenopteraMymaridae

(Yoshimoto, Kozlov & Trjapitzin, 1972)

A869CF62-F5BE-5E7B-A167-5D7A48CE4ABB

Stomarotrum prodigiosum Yoshimoto, Kozlov & Trjapitzin, 1972: 882 (original description).
Eubroncus prodigiosus (Yoshimoto, Kozlov & Trjapitzin): Triapitsyn and Berezovskiy 2002: 11–15 (taxonomic history, redescription, distribution, illustrations); Kim et al. 2017: 250–251 (taxonomic history, diagnosis, illustrations, distribution); Triapitsyn 2018: 141–142 (key, illustrations).

#### Distribution.

Palearctic region: Russia (Far East) (Yoshimoto et al. 1972; Triapitsyn and Berezovskiy 2002; Triapitsyn 2018), South Korea (Kim et al. 2017). Its record from Japan (Viggiani 2005: 72) was erroneous due to the misidentification of *E. tibetanus* Jin & Li (Triapitsyn 2018: 142).

#### Korean records.

*Eubroncus prodigiosus* (Yoshimoto, Kozlov & Trjapitzin): Kim et al. 2017: 250–251 (Gangwon-do, Gyeongsangbuk-do); Lee et al. 2021: 699 (list).

### 17. Eustochus ?atripennis

Taxon classificationAnimaliaHymenopteraMymaridae

(Curtis, 1832)

23EA1317-8D0E-5A63-BDD3-D6EAE185EBA8

Mymar atripennis Curtis, 1832: folio 411 (original description).
Eustochus atripennis (Curtis): Huber and Baquero 2007: 20–22 (taxonomic history, diagnosis, distribution).

#### Distribution.

Palearctic region: Europe; ?Japan, ?South Korea (Huber and Baquero 2007).

#### Korean records.

Eustochus ?atripennis (Curtis): Huber and Baquero 2007: 22 (Gangwon-do; possibly *E. atripennis* but F4 of the female antenna longer).

### 18. Gonatocerus cincticipitis

Taxon classificationAnimaliaHymenopteraMymaridae

Sahad, 1982

1B93808D-F866-514E-A46E-8BF1A80973CE

Gonatocerus cincticipitis Sahad, 1982: 192 (original description).
Gonatocerus (Gonatocerus) cincticipitis Sahad: Triapitsyn 2013: 15–17 (taxonomic history, distribution, redescription, diagnosis, illustrations, host).
Gonatocerus cincticipitis Sahad: Triapitsyn et al. 2021: 186–191 (taxonomic history, distribution, diagnosis, redescription, illustrations, distribution, host, molecular identification); Triapitsyn et al. 2023: 7–10 (taxonomic history, type information, distribution, revised diagnosis, redescription, illustrations, distribution, host, molecular identification).

#### Distribution.

?Oriental region: Japan (Okinawa Island) (Triapitsyn et al. 2023); Palearctic region: Japan (Sahad 1982), South Korea (Sahad and Hirashima 1984).

#### Korean records.

*Gonatocerus cincticipitis* Sahad: Sahad and Hirashima 1984: 3, 40 (Gyeonggi-do); Nam et al. 2018: 32 (Ulleungdo Island, Gyeongsangbuk-do); Lee et al. 2021: 699 (list); Triapitsyn et al. 2021: 190; Triapitsyn et al. 2023: 9; Lee et al. 2024: 132.

Gonatocerus (Gonatocerus) cincticipitis Sahad: Triapitsyn 2013: 15–16 (Gangwon-do, Gyeonggi-do).

### 19. Gonatocerus fuscicornis

Taxon classificationAnimaliaHymenopteraMymaridae

(Walker, 1846)

6599E48F-A2A3-5CBA-9A6C-2371A8411E67

Lymaenon fuscicornis Walker, 1846: 51 (original description).
Gonatocerus (Gonatocerus) fuscicornis (Walker): Triapitsyn 2013: 17–26 (taxonomic history, synonyms, type material, distribution, redescription, illustrations, diagnosis, host).
Gonatocerus fuscicornis (Walker): Triapitsyn et al. 2021: 191–193 (distribution, redescription, illustrations, diagnosis, host, molecular identification).

#### Distribution.

Afrotropical region: United Arab Emirates; Nearctic region: USA (Alaska); Oriental region: India, Taiwan (China); Palearctic region: throughout (Triapitsyn 2013, 2018; Triapitsyn et al. 2021).

#### Korean records.

Gonatocerus (Gonatocerus) fuscicornis (Walker): Triapitsyn 2013: 22–23 (Gangwon-do, Gyeonggi-do).

### 20. Gonatocerus longicornis

Taxon classificationAnimaliaHymenopteraMymaridae

Nees ab Esenbeck, 1834

0D26D008-9C7E-55D7-8DE4-368260C19BB4

Gonatocerus longicornis Nees ab Esenbeck, 1834: 193 (original description).
Gonatocerus (Gonatocerus) longicornis Nees ab Esenbeck: Triapitsyn 2013: 28–34 (taxonomic history, distribution, redescription, illustrations, diagnosis, hosts).
Gonatocerus longicornis Nees ab Esenbeck: Triapitsyn et al. 2021: 197–199 (molecular identification).

#### Distribution.

Oriental region: India, Taiwan (China), Thailand (Triapitsyn 2013, 2018); Palearctic region: throughout.

#### Korean records.

*Gonatocerus cicadellae* Nikol’skaya: Paik 1978: 180 (Gyeonggi-do: Suwon-si; host: *Cicadella viridis* (L.) [Cicadellidae]; Sahad and Hirashima 1984: 31, 34 (Gyeonggi-do).

Gonatocerus (Gonatocerus) longicornis Nees ab Esenbeck: Triapitsyn 2013: 30 (Gyeonggi-do).

*Gonatocerus longicornis* Nees: Lee et al. 2021: 699 (list).

### 21. Gonatocerus saipanensis

Taxon classificationAnimaliaHymenopteraMymaridae

(Doutt, 1955)

ABA387CE-544B-5371-B348-BEAAB4E849F3

Lymaenon saipanenis Doutt, 1955: 13 (original description).
Gonatocerus (Gonatocerus) aegyptiacus Soyka (in part, misidentification): Triapitsyn 2013: 9–13 (taxonomic history, distribution, redescription, illustrations, diagnosis, hosts).
Gonatocerus saipanensis (Doutt): Triapitsyn et al. 2021: 185–186 (illustrations), 194, 196–197 (taxonomic history, distribution, diagnosis, hosts, molecular identification).

#### Distribution.

Australasia: Australia; Oceania: American Samoa, Fiji, Northern Mariana Islands; Oriental region: China (Taiwan), India, Nepal, Pakistan, Thailand; Palearctic region: Japan, South Korea (Triapitsyn et al. 2021).

#### Korean records.

*Gonatocerus miurai* Sahad: Sahad and Hirashima 1984: 3, 36 (Gyeongsangnam-do).

*Gonatocerus aegyptiacus* Soyka: Lee et al. 2021: 699 (list).

*Gonatocerus saipanensis* (Doutt): Triapitsyn et al. 2021: 196.

### 22. Himopolynema hishimonus

Taxon classificationAnimaliaHymenopteraMymaridae

Taguchi, 1977

F1AF6D8B-10E9-550E-9D42-87B1C75CF7FD

Himopolynema hishimonus Taguchi, 1977: 137 (original description).
Himopolynema hishimonus Taguchi: Triapitsyn 2015b: 87–92 (taxonomic history, neotype designation, distribution, redescription of female, description of male, illustrations, host).

#### Distribution.

Oriental region: Bangladesh (Huber and Islam 2017), China (including Taiwan), India, Thailand (Triapitsyn 2015b); Palearctic region: Japan (Taguchi 1977), South Korea (Nam et al. 2018).

#### Korean records.

*Himopolynema hishimonus* Taguchi: Nam et al. 2018: 32 (Ulleungdo Island, Gyeongsangbuk-do); Lee et al. 2021: 699 (list); Lee et al. 2024: 132.

### 23. Litus camptopterus

Taxon classificationAnimaliaHymenopteraMymaridae

Novicky, 1953

34830EC7-A6F3-523A-BBAB-62873724CE36

Litus camptopterus Novicky, 1953: 14 (original description).
Litus camptopterus Novicky: Triapitsyn and Berezovskiy 2004b: 10–12 (taxonomic history, distribution, diagnosis, distribution, illustrations).

#### Distribution.

Nearctic region: Canada, USA; Oriental region: Taiwan (China); Palearctic region: Austria, Czechia, Kyrgyzstan, Romania, Russia, South Korea (Triapitsyn and Berezovskiy 2004b).

#### Korean records.

*Litus camptopterus* Novicky: Triapitsyn and Berezovskiy 2004b: 10, 12 (Gangwon-do).

### 24. Litus cynipseus

Taxon classificationAnimaliaHymenopteraMymaridae

Haliday, 1833

BCFE25A6-2AAC-5BF3-AC58-ADB314B28B06

Litus cynipseus Haliday, 1833: 345 (original description).
Litus cynipseus Haliday: Triapitsyn and Berezovskiy 2004b: 5–9 (taxonomic history, distribution, diagnosis, illustrations, distribution, hosts).

#### Distribution.

Nearctic region: Canada, Mexico, USA; Oriental region: India, Nepal, Taiwan (China); Palearctic region: almost throughout (Triapitsyn and Berezovskiy 2004b; Triapitsyn 2018).

#### Korean records.

*Litus cynipseus* Haliday: Triapitsyn and Berezovskiy 2004b: 7–8 (Gangwon-do).

### 25. Litus karapuz

Taxon classificationAnimaliaHymenopteraMymaridae

Triapitsyn & Berezovskiy, 2004

1A80E80B-360C-5220-B4AE-06B06BE6BFE6

Litus karapuz Triapitsyn & Berezovskiy, 2004b: 12 (original description).

#### Distribution.

Palearctic region: Japan, South Korea (Triapitsyn and Berezovskiy 2004b).

#### Korean records.

*Litus karapuz* Triapitsyn & Berezovskiy: Triapitsyn & Berezovskiy, 2004b: 12–13 (Gangwon-do, Gyeongsangnam-do [Mt. Jirisan]).

### 26. Lymaenon aureus

Taxon classificationAnimaliaHymenopteraMymaridae

(Girault, 1911)

4AD3D868-1995-5400-A74A-CB0FDE512B9D

Gonatocerus aureus Girault, 1911: 263 (original description).
Gonatocerus (Lymaenon) aureus Girault: Triapitsyn 2013: 47–56 (taxonomic history, synonymy, distribution, redescription, diagnosis, illustrations, host).
Lymaenon aureus (Girault): Huber 2015: 43.

#### Distribution.

Almost cosmopolitan (Triapitsyn 2013).

#### Korean records.

Gonatocerus (Lymaenon) aureus Girault: Triapitsyn 2013: 49, 51 (Gangwon-do, Gyeonggi-do).

#### Material examined.

South Korea • 10 ♀; Gyeongsangbuk-do, Chilgok-gun, Gisan-myeon, 35°59.07'N, 128°21.20'E, alt. 78 m, 12.x.–9.xii.2024, S.H. Kim; KNUS; • 3 ♀; Gyeongsangnam-do, Changwon-si, Masanhappo-gu, Jinjeon-myeon, 35°06.97'N, 128°24.73'E, alt. 44 m, 30.viii.–24.xi.2024, J.H. Kim; KNUS.

### 27. Lymaenon karlik

Taxon classificationAnimaliaHymenopteraMymaridae

(Triapitsyn, 2013)

CA341B5C-F573-5F97-859E-CE84A22CC731

Gonatocerus (Lymaenon) karlik Triapitsyn, 2013: 67 (original description).
Lymaenon karlik (Triapitsyn): Huber 2015: 47.

#### Distribution.

Palearctic region: China, Russia, South Korea (Triapitsyn 2013).

#### Korean records.

Gonatocerus (Lymaenon) karlik Triapitsyn: Triapitsyn 2013: 67 (Gyeonggi-do).

### 28. Lymaenon katraps

Taxon classificationAnimaliaHymenopteraMymaridae

(Triapitsyn, 2013)

65581A35-FA98-52F1-BB0D-77F8D9ECCD0E

Gonatocerus (Lymaenon) katraps Triapitsyn, 2013: 69 (original description).
Lymaenon katraps (Triapitsyn): Huber 2015: 47.

#### Distribution.

Palearctic region: Russia, South Korea (Triapitsyn 2013).

#### Korean records.

Gonatocerus (Lymaenon) katraps Triapitsyn: Triapitsyn 2013: 69 (Gyeonggi-do).

### 29. Lymaenon litoralis

Taxon classificationAnimaliaHymenopteraMymaridae

(Haliday, 1833)

C261D12D-B482-5F3D-9CFF-EAC5715BEDB9

Ooctonus litoralis Haliday, 1833: 342 (original description).
Gonatocerus (Lymaenon) litoralis (Haliday): Triapitsyn 2013: 82–97 (taxonomic history, synonymy, distribution, redescription, diagnosis, illustrations, hosts).
Lymaenon litoralis (Haliday): Huber 2015: 47.

#### Distribution.

Almost cosmopolitan (Triapitsyn 2013).

#### Korean records.

Gonatocerus (Lymaenon) litoralis (Haliday): Triapitsyn 2013: 89, 91 (Gyeonggi-do).

### 30. Mymar taprobanicum

Taxon classificationAnimaliaHymenopteraMymaridae

Ward, 1875

9C6F8000-FFC5-51A5-A0E1-B7C487BB39A6

Mymar taprobanicus Ward, 1875: 197 (original description).
Mymar taprobanicum Ward: Triapitsyn and Berezovskiy 2001: 6, 8 (illustrations), 11–12 (taxonomic history, distribution, hosts).

#### Distribution.

Almost cosmopolitan (Triapitsyn and Berezovskiy 2001).

#### Korean records.

*Mymar taprobanicum* Ward: Paik 1978: 180 (collected in rice paddy; Gyeonggi-do: Suwon-si); Lee et al. 2021: 699 (list).

#### Material examined.

South Korea – Gangwon-do • 1 ♀; Pyengchang, Jinbu, Cheokchun, 23.ix.1998, J.-Y. Choi; UCRC; • 1 ♀; Yanggu-gun: Gobangsan-ri, Dutayeon, 38°13.63'N, 127°58.93'E; alt. 328 m, 16.viii.–29.ix.2022, S.S. Buo, M.H. Kim, D.H. Park, M.C. Kim; KNA; • 1 ♀; Haean-myeon, 18.viii.2018, H.T. Shin; KNA; Gyeonggi-do: • 1 ♀ 1 ♂; Gwangju-si, Dochek-myeon, Mt. Taehwa, 5.viii.1998, J.-B. Leon, S.-H. Lee; UCRC; • 1 ♀; Gwangneung Forest, 37.7662°N, 127.1792°E, alt. 184 m, C.M. Lee: 16.v.2014, UCDC; • 1 ♀; 12.vi.2014; UCDC; • 1 ♀; 31.vii.2014; UCDC; • 1 ♀; 2.ix.2014; UCDC; • 1 ♀; 18.ix.2014; UCDC; • 1 ♀; 18.xi.2014; UCDC; • 1 ♀; 20.v.2015; UCDC; • 1 ♀; 9.ix.2015; UCDC; Pocheon-si, Soheul-eup: • 1 ♀; 37°45.04'N, 127°08.51'E, alt. 375 m; 6.iv.–4.v.2018, Kim et al.; KNA; • 1 ♀; 37°45.27'N, 127°9.96'E, alt. 102 m, 8.ix.2009, S.Y. Park et al.; KNA; • 1 ♀; 37°45.49'N, 127°10.01'E, alt. 121 m, S.Y. Park: 21.viii.2009, KNA; • 1 ♀; 15.x.2009; KNA; • 4 ♀; 15.vi.2015; KNA; • 2 ♀; 31.viii.–15.ix.2015; KNA; • 1 ♀; 30.ix.–15.x.2015; KNA; • 1 ♂; Suwon-si, Seoudun-dong, Seoul National University, 15.–17.ix.2001, J.-W. Kim; UCRC; • 6 ♀; Gyeongsangbuk-do, Chilgok-gun, Gisan-myeon, 35°59.08'N, 128°21.20'E, alt. 78 m, 12.x.–9.xii. 2024, S.H. Kim; KNUS; • 1 ♀; Gyeongsangnam-do, Changwon-si, Masanhappo-gu, Jinjeon-myeon, 35°06.98'N, 128°24.74'E, alt. 44 m, 30.viii.–24.ix.2024, J.H. Kim; KNUS.

### 31. Ooctonus us

Taxon classificationAnimaliaHymenopteraMymaridae

Triapitsyn, 2010

360FA17D-8729-52A3-9414-2742799E1274

Ooctonus us Triapitsyn, 2010: 51 (original description).

#### Distribution.

Palearctic region: Japan, South Korea (Triapitsyn 2010); this species was also tentatively identified from Taiwan (China) in the Oriental region (Triapitsyn 2018).

#### Korean records.

*Ooctonus us* Triapitsyn: Triapitsyn 2010: 51 (Gyeonggi-do).

##### Records of the fairyfly genera unidentified to species from South Korea

*Anagrus* sp(p).: Paik 1974a: 47; Paik 1978: 180, as two *Anagyrus* [sic] spp. (reared from host eggs; Gyeonggi-do: Suwon-si, Gyeongsangnam-do: Milyang-si, Jinju-si; host: *Nilaparvata lugens* [Delphacidae]); Nam et al. 2018: 32 (Ulleungdo Island, Gyeongsangbuk-do).

*Gonatocerus* sp.: Paik 1974a: 47; Kim et al. 1979: 570–579 (Gyeongsangnam-do: Jinju-si; host: *Laodelphax striatellus* [Delphacidae], *Nephotettix cincticeps* [Cicadellidae]); Chang 1980: 109–111 (Gyeongsangnam-do, Jeollanam-do; host: *Nephotettix cincticeps*); Nam et al. 2018: 32 (Ulleungdo Island, Gyeongsangbuk-do).

*Himopolynema* sp.: Nam et al. 2018: 32 (Ulleungdo Island, Gyeongsangbuk-do).

*Ooctonus* sp.: Paik 1974a: 47.

*Mymar* sp.: Paik 1974a: 47; Paik 1974b: 178.

*Polynema* sp.: Paik 1974a: 47; Paik 1978: 180 (Gyeonggi-do: Suwon-si; reared from eggs of *Hishimonus sellatus* Uhler [Cicadellidae]; thus, a very likely misidentification of *Himopolynema hishimonus*).

*Zeyanus* sp(p).: Huber 2015: 63–64.

##### Newly recorded Mymaridae from South Korea (in alphabetical order)

### 32. Mymar ermak

Taxon classificationAnimaliaHymenopteraMymaridae

Triapitsyn & Berezovskiy, 2001

566D0010-052D-54EE-8A93-60E186FDA6F5

Figs 1, 2

Mymar ermak Triapitsyn & Berezovskiy, 2001: 12 (original description).
Mymar ermak Triapitsyn & Berezovskiy: Triapitsyn 2018: 150–153 (key, illustrations).

#### Material examined.

South Korea; Gangwon-do: • 1 ♀; Goseong-gun, Bocheon-ri, Mt. Goseong, 38°21.30'N, 128°26.23'E, alt. 142 m, 11.vi.–16.vii.2022, S.S. Buo, M.H. Kim, D.H. Park, M.C. Kim; KNA; • 3 ♀ 1 ♂; Hwacheon-gun, Sangseo-myeon, Guun-ri, Mt. Baekjeok, 38°08.565'N, 127°36.383'E, alt. 384 m, 8.vi.–5.vii.2021, Y.H. Park, M.H. Kim, D.H. Park, J.Y. Kim; KNA; Yanggu-gun: • 1 ♀; Gobangsan-ri, Dutayeon, 38°13.63'N, 127°58.93'E, alt. 328 m, 9.v.–13.vi.2022, S.S. Buo, M.H. Kim, D.H. Park, M.C. Kim; KNA; • 1 ♀; Haean-myeon, Mandae-ri, Korea National Arboretum, H.T. Shin: 31.v.2014; KNA; • 1 ♀; 15.ix.2014; KNA.

**Figure 1.**
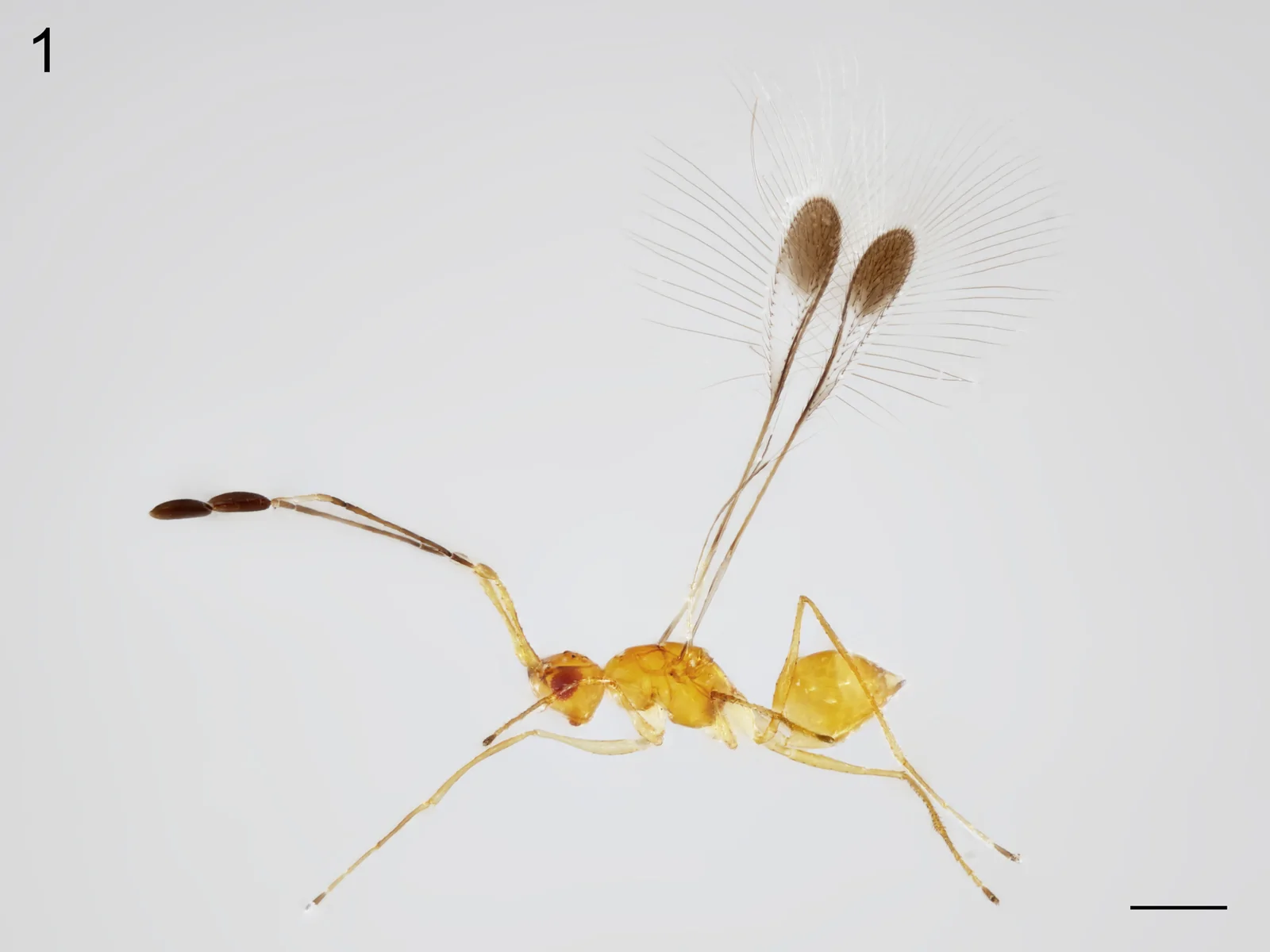
*Mymar ermak*, habitus in lateral view, female. Scale bar: 200 μm.

**Figure 2.**
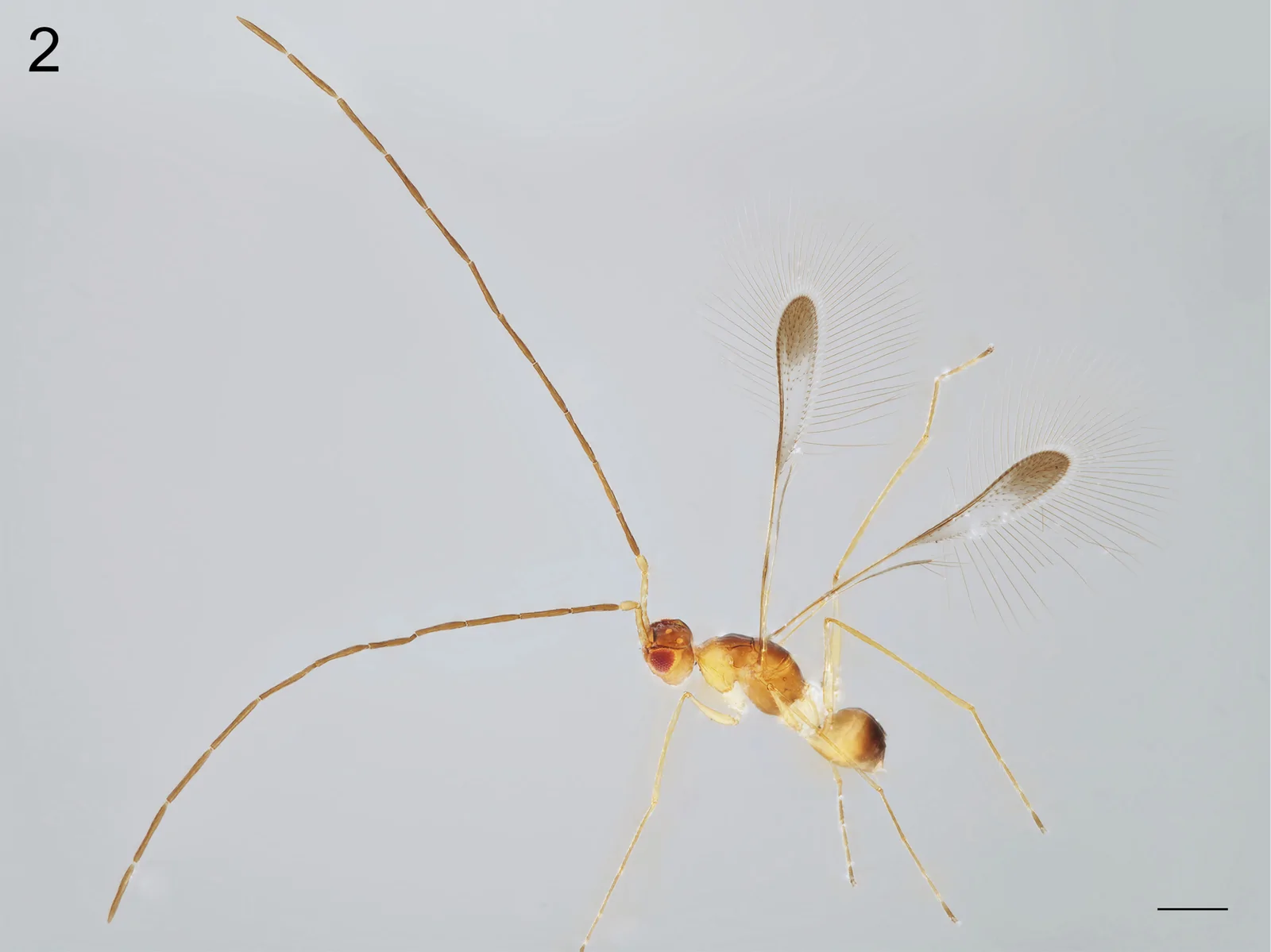
*Mymar ermak*, habitus in lateral view, male. Scale bar: 200 μm.

#### Distribution.

Palearctic region: Russia (Far East) (Triapitsyn and Berezovskiy 2001), South Korea (new record).

#### Molecular data.

GenBank accession numbers (COI): PZ377445, PZ377446, PZ377447.

### 33. Mymar maritimum

Taxon classificationAnimaliaHymenopteraMymaridae

Triapitsyn & Berezovskiy, 2001

10792C27-F06C-5933-9690-24BA3A264384

Fig. 3

Mymar maritimum Triapitsyn & Berezovskiy, 2001: 9 (original description).
Mymar maritimum Triapitsyn & Berezovskiy: Donev and Triapitsyn 2010: 66–67 (corrected diagnosis); Triapitsyn 2018: 150–153 (key, illustrations, distribution).

#### Material examined.

South Korea • 1 ♀; Gangwon-do, Yanggu-gun, Gobangsan-ri, Dutayeon, 38°13.63'N, 127°58.93'E, alt. 328 m, 16.viii.–29.ix.2022, S.S. Buo, M.H. Kim, D.H. Park, M.C. Kim; KNA.

**Figure 3.**
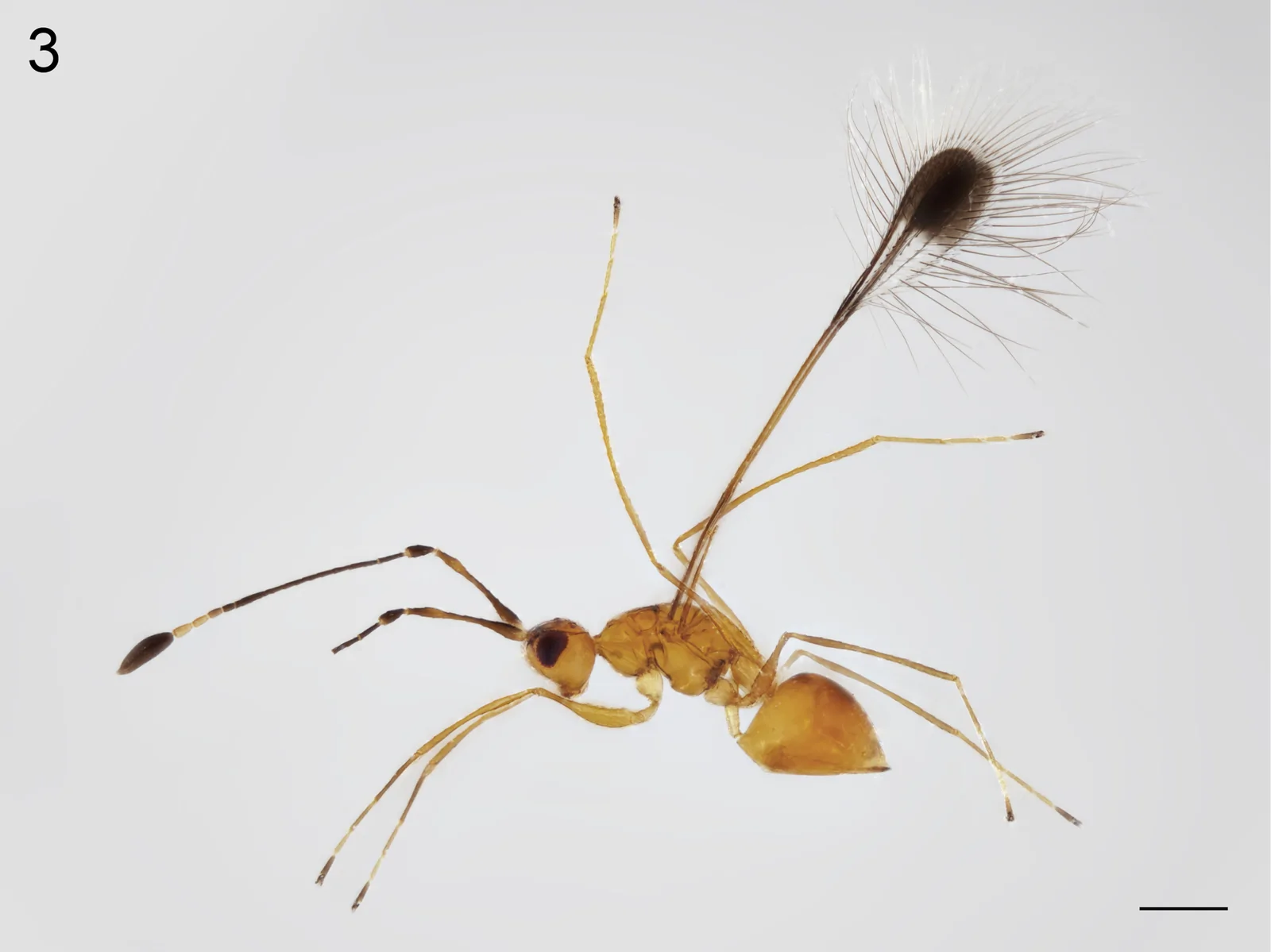
*Mymar maritimum*, habitus in lateral view, female. Scale bar: 200 μm.

#### Distribution.

Oriental region: Taiwan (China) (Triapitsyn 2018); Palearctic region: Russia (Far East) (Triapitsyn and Berezovskiy 2001), South Korea (new record).

#### Molecular data.

GenBank accession number (COI): PZ377449.

### 34. Mymar pulchellum

Taxon classificationAnimaliaHymenopteraMymaridae

Curtis, 1832

23C64F7B-A51F-53B3-9B0B-52922BCA2963

Fig. 4

Mymar pulchellus Curtis, 1832: folio 411.
Mymar pulchellum Curtis: Triapitsyn and Berezovskiy 2001: 6–9 (illustration, key, taxonomic history, distribution); Triapitsyn 2018: 150 (key).

#### Material examined.

South Korea • 1 ♀; Gangwon-do, Yanggu-gun, Gobangsan-ri, Dutayeon, 38°13.63'N, 127°58.93'E, alt. 328 m, 16.viii.–29.ix.2022, S.S. Buo, M.H. Kim, D.H. Park, M.C. Kim; KNA.

**Figure 4.**
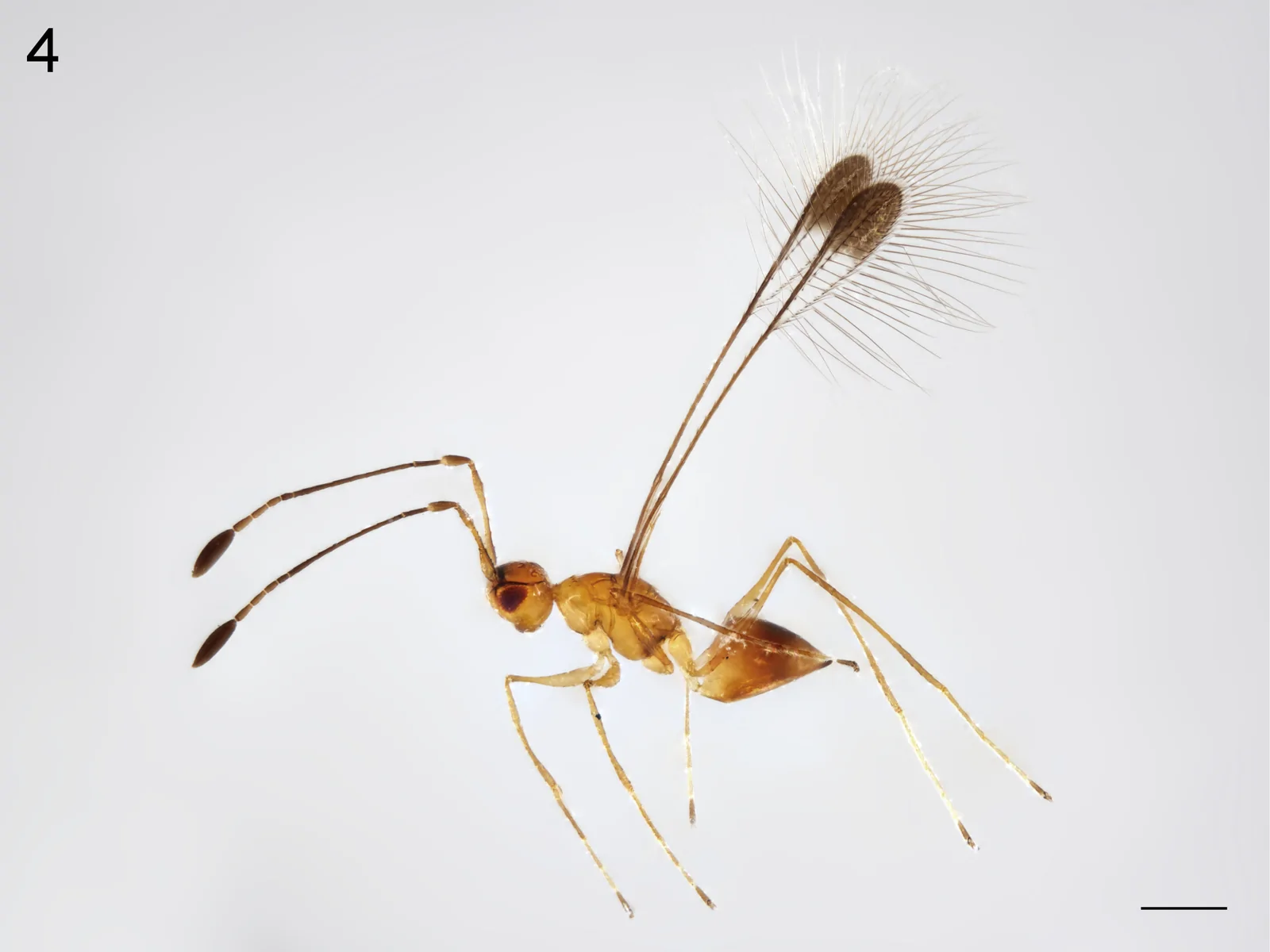
*Mymar pulchellum*, habitus in lateral view, female. Scale bar: 200 μm.

#### Distribution.

Palearctic region (Triapitsyn and Berezovskiy 2001) including South Korea (new record). Records of *M. pulchellum* from other biogeographical regions need confirmation using molecular methods.

#### Molecular data.

GenBank accession number (COI): PZ377448.

### 35. Zeyanus knuz

Taxon classificationAnimaliaHymenopteraMymaridae

Triapitsyn & Kim sp. nov.

1A41D7D9-DD1D-5A8D-A28D-56D301427A8C

https://zoobank.org/D46FCBA3-AA5D-479E-B17D-A31E0798B43B

Figs 5, 6, 7, 8, 9, 10, 11, 12

#### Type material.

***Holotype***. Japan • ♀; Honshu Island, Aichi Prefecture, Asahi, Yawata, alt. 650 m, 15–25.ix.1998, M. Ozawa, deciduous forest; ZLMU; on slide (Fig. 6). ***Paratypes***. Japan • 1 ♀; Honshu Island, Gifu Prefecture, Kani, Katabira, 2.–8.x.2004, K. Yamagishi; ZLMU; on point (Fig. 11). South Korea • 2 ♀; Gyeonggi-do, Pocheon-si, Soheul-eup, Gwangneung Forest, 37.7662°N, 127.1792°E, alt. 174 m, 11.vii.2014, C.M. Lee; UCDC, UCRC; on points (Fig. 12).

#### Diagnosis.

In Triapitsyn et al. (2017: 158), *Z. knuz* keys to couplet 5, together with *Z. longiterebratus* (Subba Rao) and *Z. kweishuilini* Triapitsyn. The former species is known only from India, and the latter is known only from southern Taiwan (China), both in the Oriental region (Triapitsyn et al. 2017). The female of *Z. knuz* differs from that of *Z. kweishuilini*, to which it is most similar, by the morphological features indicated in the key below.

#### Description.

**Figure 5.**
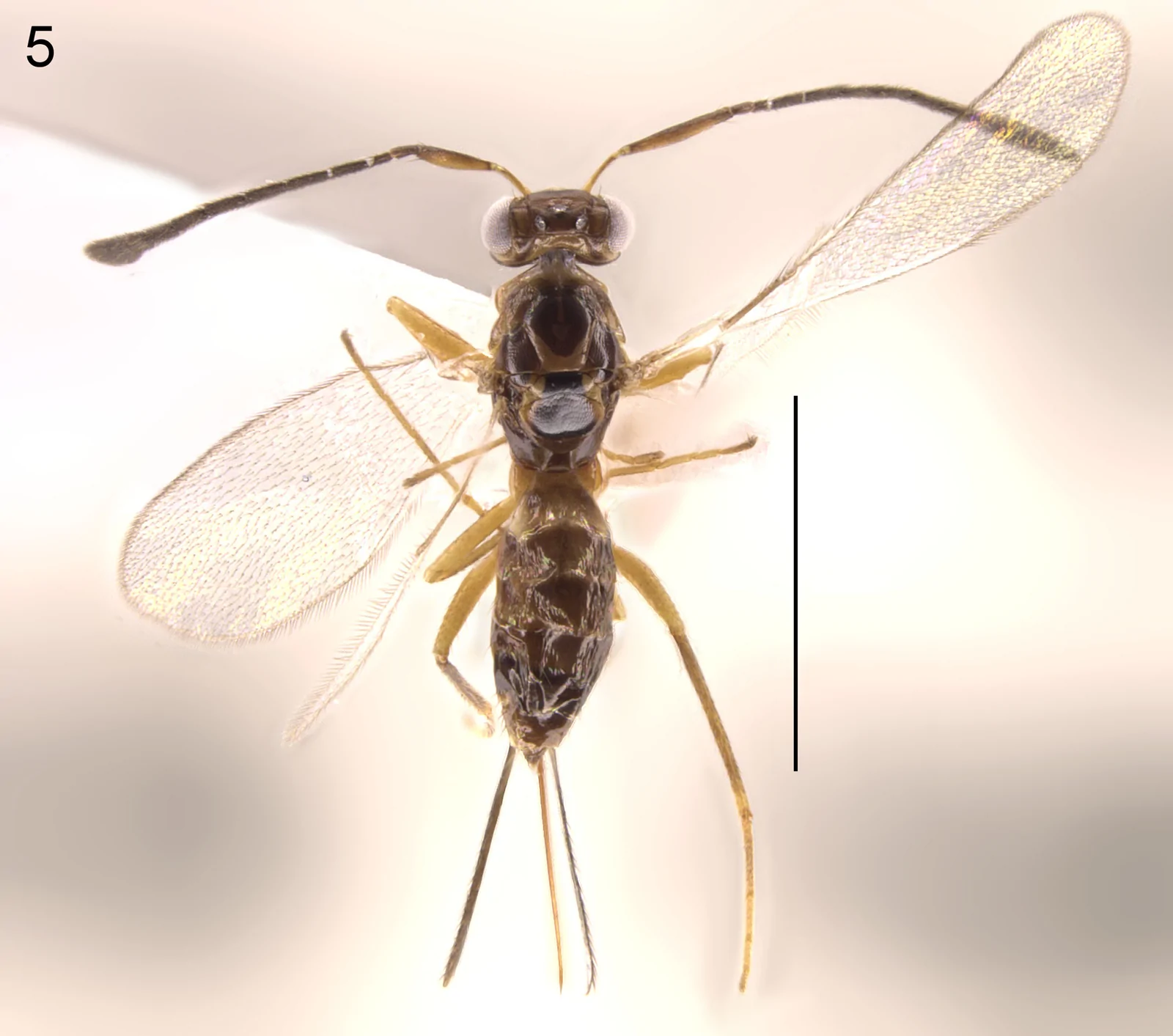
*Zeyanus knuz* sp. nov., female, holotype: habitus of the dry specimen prior to slide-mounting. Scale bar: 1 mm.

Female (holotype, Figs 5, 7–10). Body (Figs 5, 8) mostly brown to dark brown with patches of yellow on mesoscutum and scutellum, and pale brown on head, propodeum, and base of gaster; ovipositor sheaths brown; radicle pale brown, remainder of scape brown, and pedicel and flagellum dark brown; legs brownish-yellow.

***Antenna*** (Fig. 7). Radicle 0.33× total length of scape, remainder of scape 4.5× as long as wide; pedicel a little longer than F1; F1 the shortest funicle segment and notably longer than wide, F2, F6, and F7 equal in length and a little shorter than F3, F4 and F5 (the longest funiculars), F8 slightly shorter than F7 and the widest funicular; mps on F1 (1), F2 (2), F3 (2), F4 (2), F5 (3), F6 (3), F7 (3), and F8 (4); clava with 17 mps, 4.8× as long as wide, notably longer than combined length of F6–F8 (almost as long as combined length of F5–F8).

**Figures 6–10.**
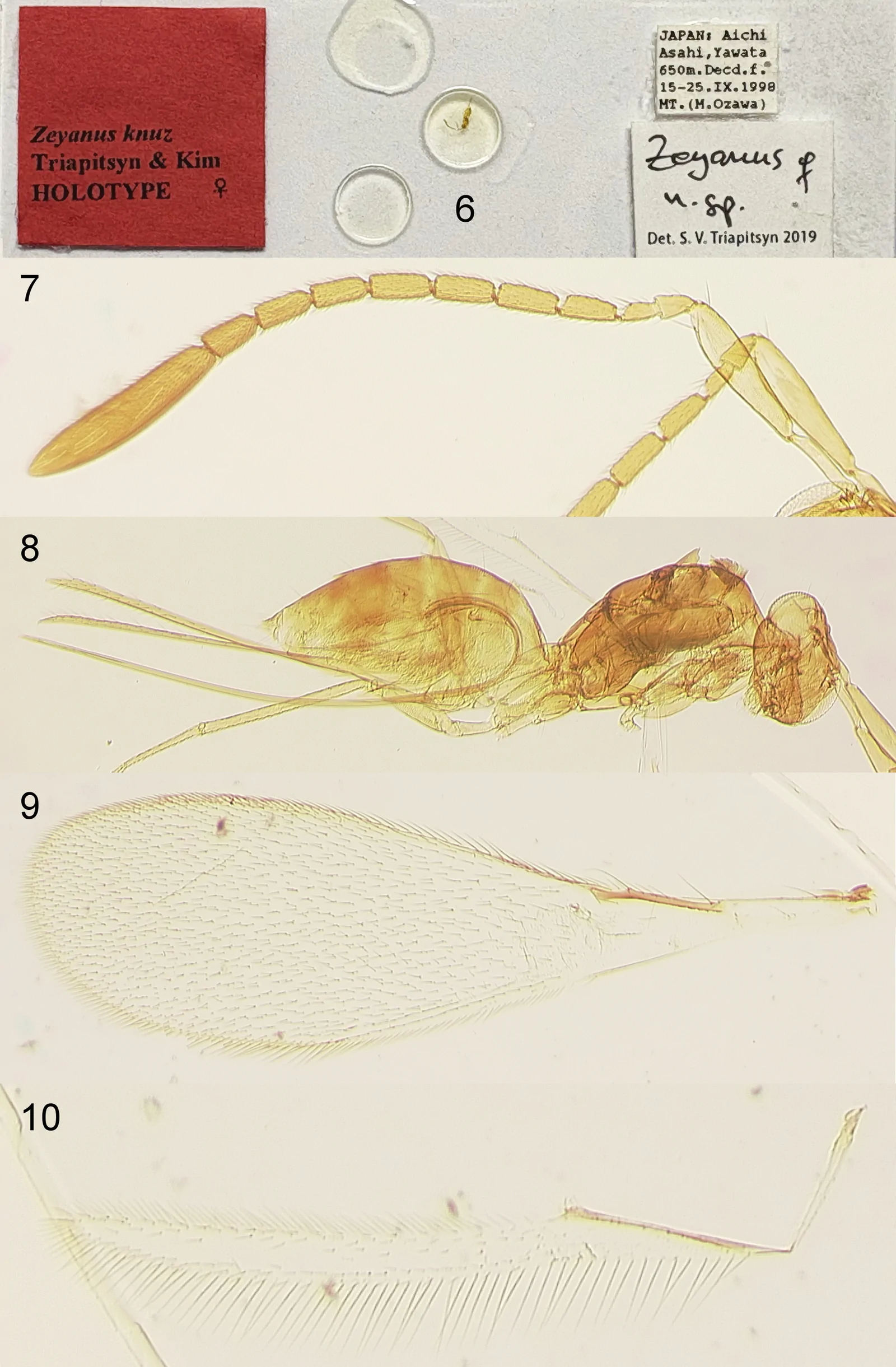
*Zeyanus knuz* sp. nov., female, holotype: **6**. Slide; **7**. Antenna; **8**. Body; **9**. Fore wing; **10**. Hind wing.

***Mesosoma*** (Fig. 8). Shorter than gaster; midlobe of mesoscutum with a pair of adnotaular setae.

***Wings*** (Fig. 9). Fore wing 3.3× as long as wide; longest marginal seta 0.16× maximum wing width; disc almost hyaline, bare behind submarginal vein and base of marginal vein, cubital row of microtrichia behind marginal vein absent or incomplete, with only a few (3 or 4) microtrichia present behind apex of marginal vein and stigmal vein. Hind wing (Fig. 10) 18.4× as long as wide; disc mostly bare (except for admarginal rows of setae and some additional setae) and hyaline; longest marginal seta 1.7× maximum wing width.

***Metasoma*** (Fig. 8). Ovipositor occupying almost 0.9× length of gaster, exserted beyond its apex by 0.46× its total length; ovipositor length: metatibia length ratio 2.8: 1.

***Measurements* (µm) of the holotype (as length or length: width)**. Body (of the dry-mounted specimen prior to slide-mounting, Fig. 5): 1750, and of the slide-mounted specimen 2005; head (of the dry-mounted specimen prior to slide-mounting) 215; mesosoma 736; petiole 43; gaster 984; ovipositor 1747. Antenna: radicle 182; remainder of scape 364; pedicel 85; F1 73; F2 121; F3 130; F4 130; F5 130; F6 121; F7 121; F8 115; clava 476. Fore wing 2017:614; longest marginal seta 100. Hind wing 1328:72; longest marginal seta 123.

**Figures 11, 12.**
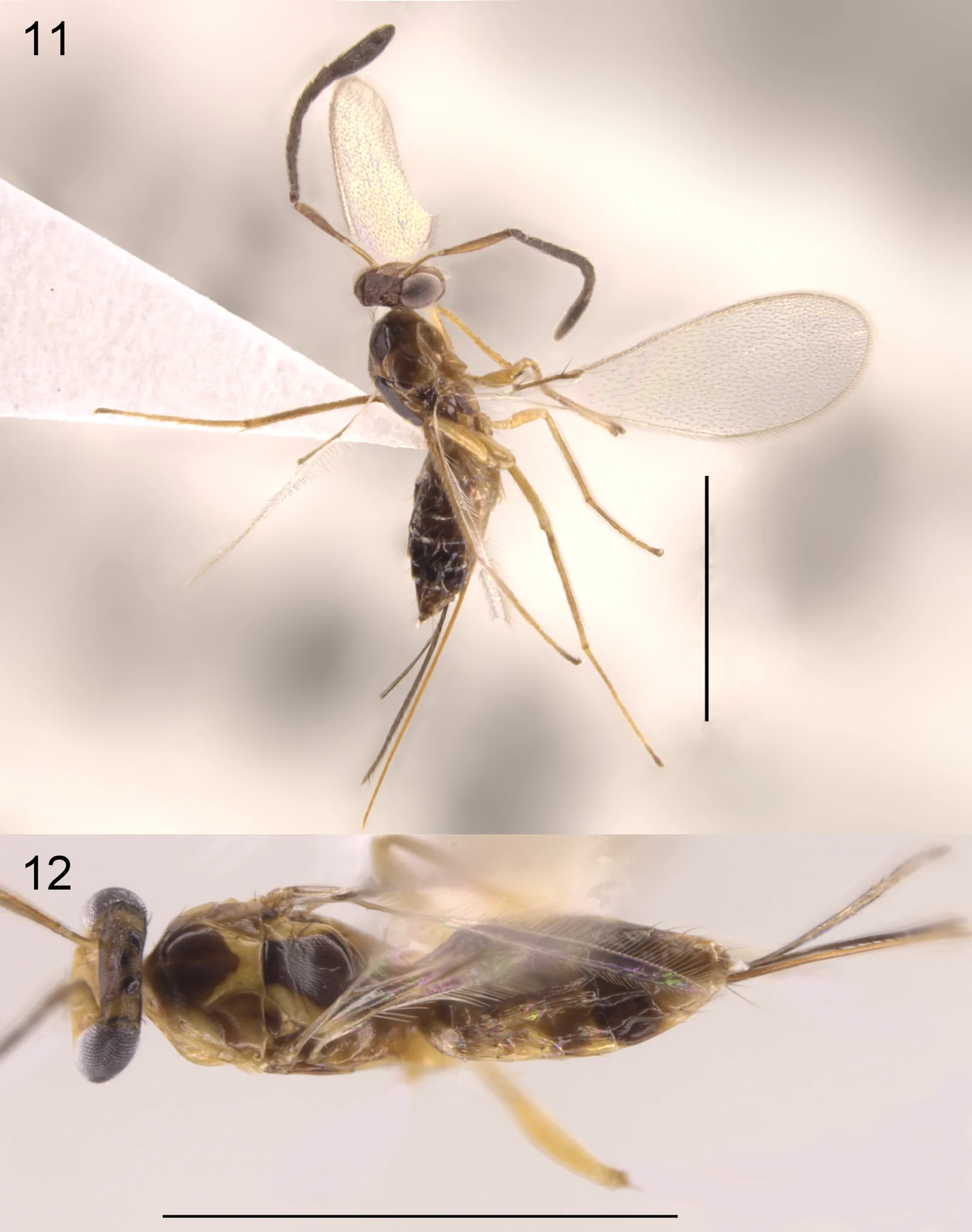
*Zeyanus knuz* sp. nov., female paratypes, habitus: **11**. Specimen from Katabira, Kani, Gifu Prefecture, Honshu Island, Japan; **12**. Specimen from Gwangneung Forest, Gyeonggi-do, South Korea. Scale bar: 1 mm.

***Variation* (paratypes)**. Body length of dry-mounted specimens 1340–1420 µm (Figs 11, 12). In some specimens, body sometimes with more pronounced yellow or pale brown patches (Fig. 12).

**Male**. Unknown.

#### Etymology.

The species name is a noun in apposition and a combination of letters without any meaning.

#### Distribution.

Palearctic region: Japan, South Korea.

#### Hosts.

Unknown.

### 36. Zeyanus sunayez

Taxon classificationAnimaliaHymenopteraMymaridae

Triapitsyn & Kim sp. nov.

E7D8CAAE-94FA-5ADB-B897-54596710A113

https://zoobank.org/6F37E4F3-286A-4ABA-BCD1-C6132A1AD95C

Figs 13, 14, 15, 16, 17, 18, 19, 20

#### Type material.

**Figures 13–17.**
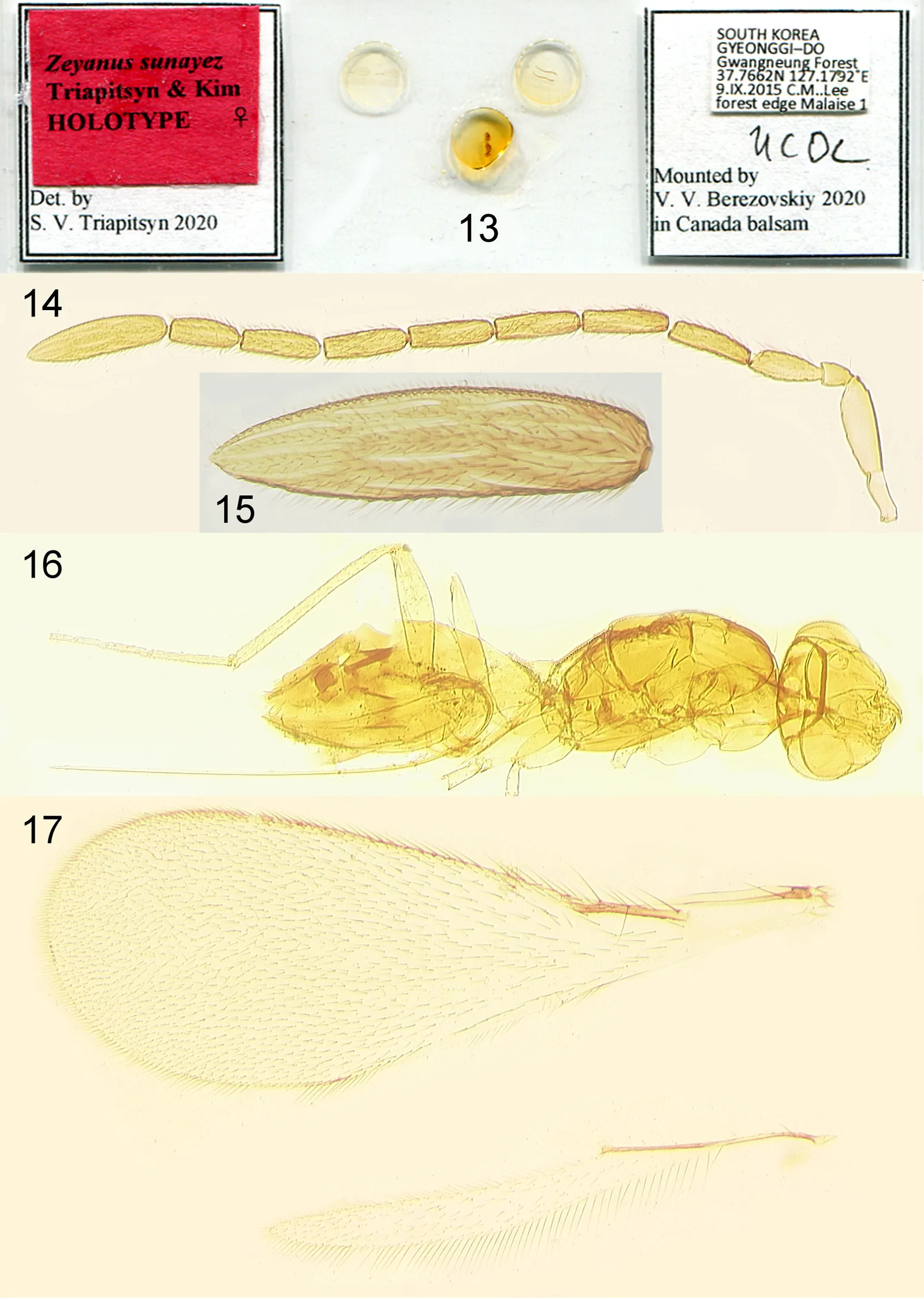
*Zeyanus sunayez* sp. nov., female, holotype: **13**. Slide; **14**. Antenna; **15**. Clava; **16**. Body; **17**. Fore and hind wings.

***Holotype***. South Korea • ♀; Gyeonggi-do, Pocheon-si, Soheul-eup, Gwangneung Forest, 37.7662°N, 127.1792°E, alt. 174 m, 9.viii. 2015, C.M. Lee, forest edge, Malaise trap; NIBR; on slide (Fig. 13). ***Paratypes***. Japan • 1 ♂; Ryukyu Islands, Kagoshima Prefecture, Amami Island (Amami Oshima), Mt. Yui, alt. 400 m, 15.v.–21.vi.2001, T. Muroi, Y. Maeda; ZLMU; on point (Fig. 21); South Korea: Gangwon-do: Yanggu-gun: • 1 ♀; Haean-myeon, Mandae-ri, Korea National Arboretum, 38°15.16'N, 128°06.71'E, alt. 639 m, I. Kim: 15.–30.vi.2020, KNA; in ethanol; • 1 ♀; 7.–31.x.2020; KNA; in ethanol; • 2 ♀; Gobangsan-ri, Dutayeon, 38°13.63'N, 127°58.93'E, alt. 328 m, 11.vii.–16.viii.2022, S.S. Buo, M.H. Kim, D.H. Park, M.C. Kim; KNA; in ethanol; • 1 ♀; Haean-myeon, Mandae-ri, Korea National Arboretum, 15.vii.2014, H.T. Shin; KNA; on point; Gyeonggi-do: • 2 ♀; same locality and collector as holotype; UCDC, UCRC; on points; • 2 ♀; Namyangju-si, Jinjeop-eup, Bupyeong-ri, 37°45.17'N, 127°09.07'E, alt. 395 m, 29.vi.–16.vii.2018, M.H. Kim, M.C. Kim, H.O. Kim, K.A. Park; KNA; in ethanol; Paju-si: • 1 ♀; Beobwon-eup, Ungdam-ri, Mt. Papyyeong, 37°54.62'N, 126°52.89'E, alt. 306 m, 8.ix.–6.x.2021, Y.H. Park, M.H. Kim, D.H. Park, J.Y. Kim; KNA • 1 ♀; Papyeong-myeon, Neulno-ri, Mt. Papyyeong, 37°54.62'N, 126°51.98'E, alt. 260 m, 11.vii.–16.viii.2022, S.S. Buo, M.H. Kim, D.H. Park, M.C. Kim; KNA; in ethanol; Pocheon-si, Soheul-eup: • 2 ♀; 37°45.20'N, 127°08.58'E, alt. 374 m, Kim, Kim, J.W. Nam: 30.vi.–17.vii.2017; KNA; on points; • 1 ♀; 31.vii.–16.viii.2017; KNA; on point; • 1 ♀; 37°45.49'N, 127°10.01'E, alt. 121 m, Park, Choi, Nam, Shin, Kim: 29.vi.2015, KNA; on point; • 1 ♀; 31.vii.–17.viii.2015; KNA; on point; • 1 ♀; 37°54.62'N, 126°52.88'E, alt. 304 m, 8.ix.–6.x.2021, Y.H. Park, M.H. Kim, D.H. Park, J.Y. Kim; KNA; in ethanol; • 2 ♀; Uijeongbu-si, Milak-dong, Soribong Peak, 37°45.02'N, 127°08.54'E, alt. 382 m, M.H. Kim, M.C. Kim, H.O. Kim, K.A. Park: 29.vi.–16.vii.2018; KNA; in ethanol; • 1 ♀; 16.–30.vii.2018; KNA; in ethanol; • 1 ♀; Gyeongsangbuk-do, Seokpo-myeon, Bonghwa, 37°04.53'N, 129°05.22'E, alt. 614 m, 16.viii.2016, J.W. Nam, Kim, Kim, Park; KNA; on point; • 1 ♀; Gyongsangnam-do, Geoje-si, Dundeok-myeon, Bangha-ri, 34°51.20'N, 128°32.40'E, alt. 172 m, 21.vi.–19.vii.2022, S.S. Uh, M.H. Kim, M.C. Kim, D.H. Park; KNA; in ethanol.

#### Diagnosis.

In Triapitsyn et al. (2017: 157), *Z. sunayez* keys to couplet 2, together with *Z. asulcifrons* (Zeya), originally described from India (Zeya and Hayat 1995) and also known from China and Thailand in the Oriental region (Triapitsyn et al. 2017). Both taxa have a cubital row of setae behind the marginal vein of the fore wing complete and a few additional microtrichia behind the marginal vein (as in Fig. 17). In addition to the diagnostic features indicated in the key below, funicle segments of the female antenna of *Z. asulcifrons* are relatively shorter (Zeya and Hayat 1995: 133, fig. 18; Triapitsyn et al. 2017: 149, 150, figs 3A, 4A) than in *Z. sunayez* (Fig. 14).

#### Description.

Female (holotype, Figs 13–17). Head mostly pale brown except most of vertex brown to dark brown; remainder of body (Fig. 16) mostly brown to dark brown with patches of pale brown posteriorly on midlobe of mesoscutum, propodeum and entire base of gaster; antenna brown to dark brown except scape, pedicel and F1 paler; legs brownish-yellow.

***Antenna*** (Fig. 14). Radicle 0.34× total length of scape, remainder of scape 3.0× as long as wide; pedicel much shorter than F1; F1 the shortest funicle segment and much longer than wide, F2–F6 subequal in length (F3–F5 the longest), F7 and F8 progressively shorter; mps on F1 (5), F2 (6), F3 (5 or 6), F4 (8), F5 (8), F6 (8), F7 (8), and F8 (8); clava (Fig. 15) apparently with 21 mps, 3.9× as long as wide, almost as long as combined length of F7 and F8 (slightly shorter).

**Figure 18.**
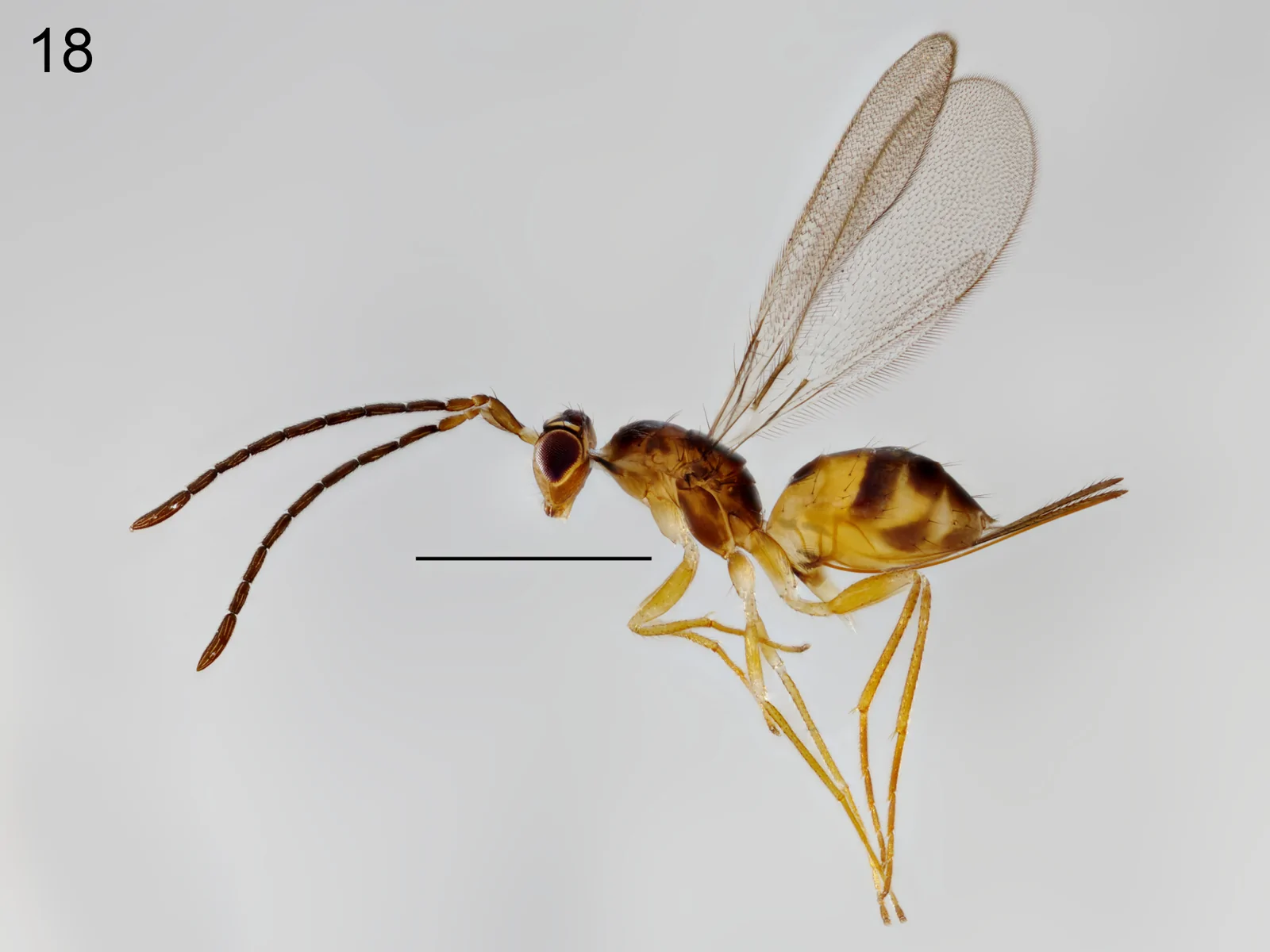
*Zeyanus sunayez* sp. nov., female, paratype from Gyongsangnam-do, Geoje-si, Dundeok-myeon, Bangha-ri: habitus. Scale bar: 1 mm.

***Mesosoma*** (Fig. 16). Shorter than gaster (as in Fig. 18). Midlobe of mesoscutum with a pair of adnotaular setae.

***Wings*** (Fig. 17). Fore wing 2.8× as long as wide; longest marginal seta 0.12× maximum wing width; disc almost hyaline, bare behind submarginal vein and microtrichia sparse behind marginal and stigmal veins; cubital line of microtrichia complete. Hind wing (Fig. 17) wide, 12.1× as long as wide; disc hyaline, with microtrichia behind and beyond apex of venation; longest marginal seta slightly longer than maximum wing width.

***Metasoma*** (Fig. 16). Ovipositor occupying ~ 0.8× length of gaster, exserted beyond its apex by 0.45× its total length; ovipositor length: metatibia length ratio 2.2: 1.

***Measurements* (µm) of the holotype (as length or length: width)**. Body (of the dry-mounted specimen prior to slide-mounting): 1866, and of the slide-mounted specimen 2091; head 370; mesosoma 750; petiole 30; gaster 959; ovipositor 1501 (at least). Antenna: radicle 103; remainder of scape 212; pedicel 66; F1 142; F2 179; F3 182; F4 182; F5 182; F6 179; F7 173; F8 148; clava 306. Fore wing 2005:713; longest marginal seta 85. Hind wing 1501:124; longest marginal seta 131.

**Figures 19, 20.**
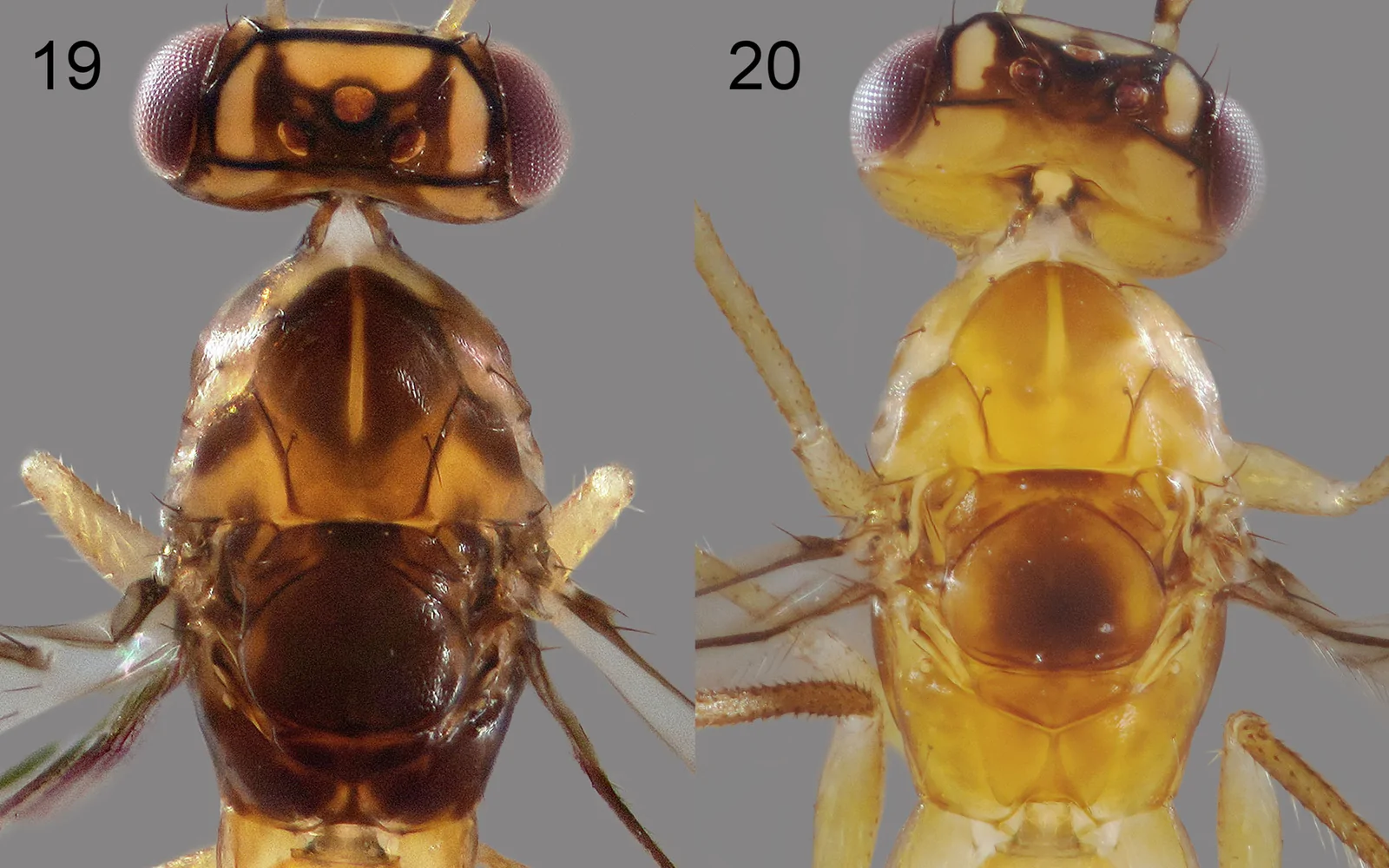
Color variation of *Zeyanus sunayez* sp. nov., mesosoma with pale brown patches (**19**) or mostly yellow (**20**).

***Variation* (paratypes)**. Body length of dry-mounted specimens 1540–1907 µm, and of specimens in ethanol 1683–1997 µm (Fig. 18). In some specimens, mesosoma with pale brown patches (Fig. 19) or mostly yellow (Fig. 20).

**Figures 21, 22.**
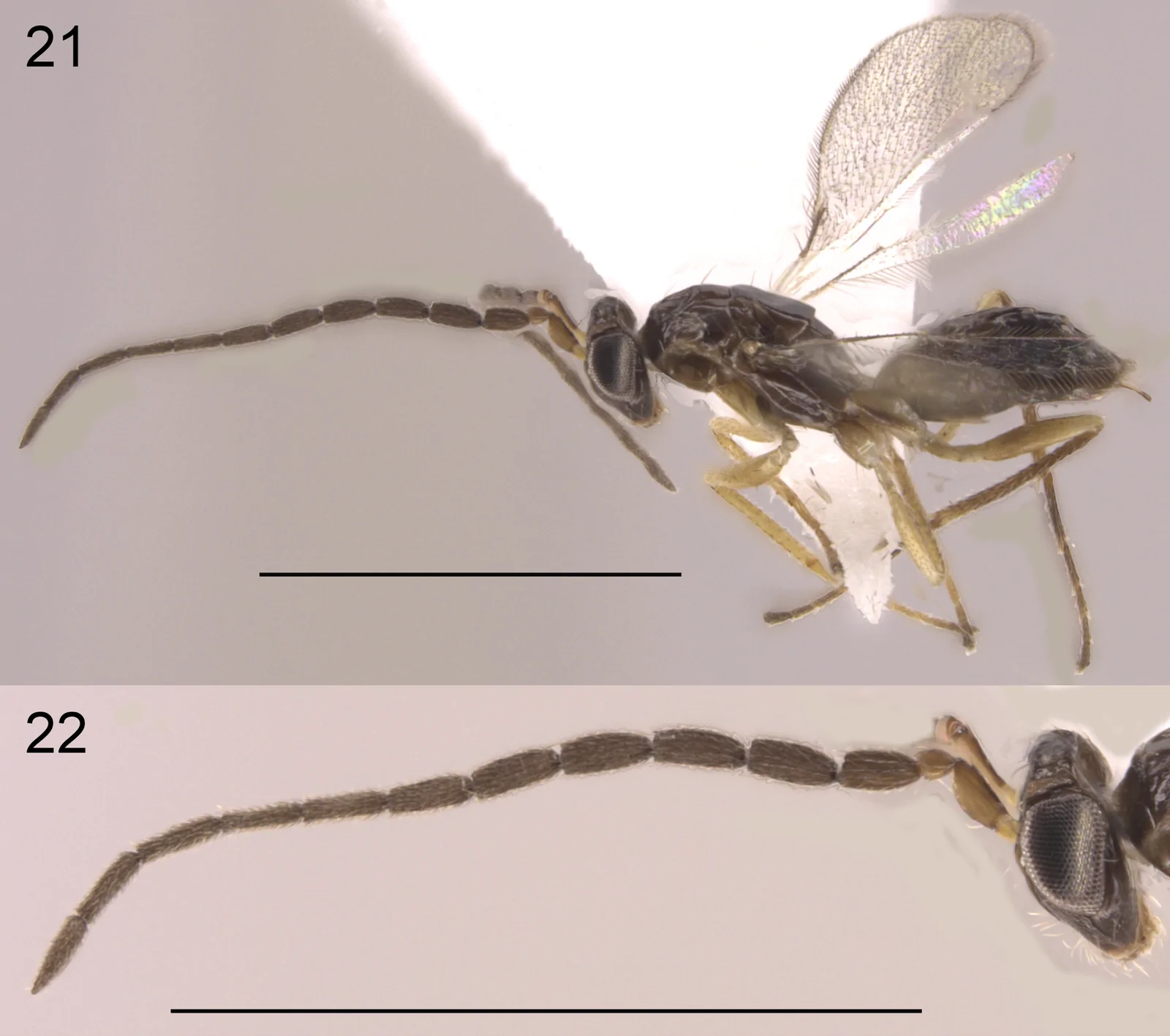
*Zeyanus sunayez* sp. nov., male paratype from Amami Island, Ryukyu Islands, Japan: **21**. Habitus in lateral view; **22**. Antenna. Scale bar: 1 mm.

**Male** (paratype, Figs 21, 22). Body length 1453 µm (dry-mounted specimen, Fig. 21). Body notably darker than female, almost uniformly brown to dark brown; antenna brown to dark brown, with scape and pedicel paler. Otherwise similar to female except for the normal sexually dimorphic features of antenna and genitalia. Antenna (Fig. 22) with scape ~ 2.5× as long as wide.

#### Etymology.

The species name is a noun in apposition and a reverse spelling of the genus name.

#### Distribution.

Oriental region: Japan (Amami Island, Ryukyu Islands); Palearctic region: South Korea.

#### Hosts.

Unknown.

#### Molecular data.

GenBank accession number (COI): PZ653912.

#### Remarks.

Huber (2015) reclassified the *Gonatocerus* group of genera (now classified in the tribe Gonatocerini) and separated *Zeyanus* from *Gonatocerus*. Zeya (2014) provided a key to the six Indian species of *Zeyanus*, which were treated then in the informal *asulcifrons* species group of *Gonatocerus*. Triapitsyn et al. (2017) described two species of *Zeyanus* from Taiwan (China) and gave a key to the 11 valid species of the genus, all from the Oriental region (India and Taiwan (China)). *Zeyanus* occurs in the Afrotropical, Oriental, and eastern Palearctic regions (Huber 2015). The specimens of *Zeyanus* from Japan and South Korea mentioned in Huber (2015), without providing any other data on them, very likely belong either to *Z. knuz* or *Z. sunayez*, or both.

The only host known so far for the genus is of an unidentified *Zeyanus* sp. reared in Chonburi Province of Thailand from a *Ledra* sp. (Cicadellidae: Ledrinae) attacking chili, *Capsicum* sp. (Solanaceae) (Triapitsyn et al. 2017). As noted by them, Ledrinae include some very large leafhoppers and that correlates quite well with rather large (for fairyflies) body size of *Zeyanus* species. Further rearings from Ledrinae would confirm this.

##### Key to species of *Zeyanus* (females)

**Table:** 

1	Head and mesosoma pale yellow or yellowish-brown to pale brown, with brown to dark brown patch(es) on mesoscutum and/or scutellum	**2**
–	Head and mesosoma completely brown to black, without yellow or pale brown patches	**8**
2 (1)	Fore wing disc behind marginal vein with cubital row of microtrichia complete (as in Fig. 17)	**3**
–	Fore wing disc behind marginal vein with cubital row of microtrichia absent or incomplete (as in Fig. 9)	**4**
3 (2)	F1 and F2 with at most 3 mps; clava approx. as long as combined length of F6–F8; gaster yellow, with dark cross-bands	***Z. asulcifrons* (Zeya)**
–	F1 with 5, F2 with 6 mps; clava almost as long as combined length of F7 and F8 (Fig. 14); gaster mostly dark except notably paler basally (Fig. 16)	***Z. sunayez* Triapitsyn & Kim, sp. nov**.
4 (2)	Flagellum with F6 and F7 yellowish-white	***Z. bialbifuniculatus* (Subba Rao)**
–	Flagellum with F6 and F7 brown to dark brown	**5**
5 (4)	F1 much shorter than F2	**6**
–	F1 approx. as long as F2	***Z. rileyi* (Zeya)**
6 (5)	F1 approx. as long as wide	***Z. longiterebratus* (Subba Rao)**
–	F1 at least twice as long as wide	**7**
7 (6)	F1 with 1 mps; clava 4.8× as long as wide and with 17 mps; fore wing disc with at most 4 microtrichia behind apex of marginal and stigmal veins and not close to posterior margin (Fig. 9)	***Z. knuz* Triapitsyn & Kim, sp. nov**.
–	F1 without mps; clava 4.0× as long as wide and with 21 mps; fore wing disc with at least 14 microtrichia behind apex of marginal vein and closer to posterior margin	***Z. kweishuilini* Triapitsyn**
8 (1)	Flagellum with F6 and F7 brown to dark brown	**9**
–	Flagellum with F6 and F7 yellowish-white	***Z. fulvipodus* (Subba Rao)**
9 (8)	Fore wing disc behind marginal vein with a few microtrichia	**12**
–	Fore wing disc behind marginal vein bare	**10**
10 (9)	Pedicel approx. as long as F1; procoxa dark brown or black, profemur brown to dark brown	**11**
–	Pedicel ~ 0.6× as long as F1; procoxa and profemur brownish-yellow	***Z. sergueii* (Manickavasagam & Rameshkumar)**
11 (10)	Head black; clava as long as combined length of F4–F8	***Z. unicolouratus* (Subba Rao)**
–	Head brown; clava as long as combined length of F5–F8	***Z. shihi* Triapitsyn**
12 (9)	F1 without mps, F2 and F3 each with 1 mps, F4–F8 each with 2 mps; ovipositor ~ 2.3× as long as mesotibia	***Z. devikulamus* (Mani & Saraswat)**
–	F1 with 3 mps, F2 and F3 each with at least 4 mps, F4–F8 each with at least 5 mps; ovipositor ~ 2.9× as long as mesotibia	***Z. jaintiacus* (Zeya)**

## Supplementary Material

XML Treatment for Anagroidea marina

XML Treatment for Anagrus (Anagrus) arboridiae

XML Treatment for Anagrus (Anagrus) atomus

XML Treatment for Anagrus (Anagrus) flaviapex

XML Treatment for Anagrus (Anagrus) hirashimai

XML Treatment for Anagrus (Anagrus) incarnatus

XML Treatment for Anagrus (Anagrus) obscurus

XML Treatment for Anagrus (Paranagrus) optabilis

XML Treatment for Anagrus (Anagrus) striatus

XML Treatment for Anaphes (Patasson) collinus

XML Treatment for Anaphes (Anaphes) flavipes

XML Treatment for Anaphes (Anaphes) gauthieri

XML Treatment for Anaphes (Anaphes) regulus

XML Treatment for Dicopomorpha koreana

XML Treatment for Erythmelus (Erythmelus) nuinu

XML Treatment for Eubroncus prodigiosus

XML Treatment for Eustochus ?atripennis

XML Treatment for Gonatocerus cincticipitis

XML Treatment for Gonatocerus fuscicornis

XML Treatment for Gonatocerus longicornis

XML Treatment for Gonatocerus saipanensis

XML Treatment for Himopolynema hishimonus

XML Treatment for Litus camptopterus

XML Treatment for Litus cynipseus

XML Treatment for Litus karapuz

XML Treatment for Lymaenon aureus

XML Treatment for Lymaenon karlik

XML Treatment for Lymaenon katraps

XML Treatment for Lymaenon litoralis

XML Treatment for Mymar taprobanicum

XML Treatment for Ooctonus us

XML Treatment for Mymar ermak

XML Treatment for Mymar maritimum

XML Treatment for Mymar pulchellum

XML Treatment for Zeyanus knuz

XML Treatment for Zeyanus sunayez

## Additional information

### Artificial Intelligence (AI) use

The authors accept full responsibility for the content of the manuscript, including the disclosure of any use of AI.

No AI tools were used in the preparation of this manuscript.

### Author ORCIDs

S.V. Triapitsyn https://orcid.org/0000-0002-5086-7847

S. Kim https://orcid.org/0009-0005-5378-0447

I. Kang https://orcid.org/0000-0002-8501-1758

### Data availability

All of the data that support the findings of this study are available in the main text.
